# CPTAC Consensus Subtypes Add No Incremental Prognostic Value Beyond Stage and Grade in Clear Cell Renal Cell Carcinoma: A Proteomic Reanalysis

**DOI:** 10.3390/life16101752

**Published:** 2026-10-19

**Authors:** Seyma Yasar, Fatma Hilal Yagin, Sarah A. Alzakari, Amal K. Alkhalifa, Fahaid Al-Hashem, Abedelmalek Kalefh Tabnjh

**Affiliations:** 1 Department of Biostatistics and Medical Informatics, Faculty of Medicine, Inonu University, Malatya 44280, Türkiye; seyma.yasar@inonu.edu.tr; 2 Department of Biostatistics and Medical Informatics, Faculty of Medicine, Balikesir University, Balikesir 10145, Türkiye; 3 Department of Computer Sciences, College of Computer and Information Sciences, Princess Nourah bint Abdulrahman University, Riyadh 11671, Saudi Arabia; saalzakari@pnu.edu.sa (S.A.A.); akalkalifh@pnu.edu.sa (A.K.A.); 4 Department of Physiology, College of Medicine, King Khalid University, Abha 61421, Saudi Arabia; fahaid999@yahoo.com; 5 Department of Cariology, Institute of Odontology, the Sahlgrenska Academy, University of Gothenburg, 40530 Gothenburg, Sweden; 6 Department of Applied Dental Sciences, Faculty of Applied Medical Sciences, Jordan University of Science and Technology, Irbid 22110, Jordan; 7 Department of Pathology, Saveetha Medical College and Hospital, Saveetha Institute of Medical and Technical Sciences, Chennai 602105, Tamil Nadu, India

**Keywords:** clear cell renal cell carcinoma, molecular subtypes, incremental prognostic value, risk stratification, proteomics, machine learning, null distribution

## Abstract

Molecular subtypes from multi-omic profiling have refined the biological characterization of clear cell renal cell carcinoma (ccRCC), but whether they add prognostic information to clinicopathological variables already in use is unclear. We evaluated the incremental prognostic value of proteomically assigned CPTAC consensus subtypes (NMF1–NMF3) against stage and grade, and asked whether subtype identity is carried by a biologically specific set of proteins. Subtypes were predicted from tumor proteomes in 211 patients under nested cross-validation and a locked hold-out design, and the analysis was repeated using methylation subtypes defined without proteomic input. A transcriptome classifier was transferred to 533 TCGA-KIRC tumors, and the transferred subtypes entered nested Cox models containing age, stage and Fuhrman grade. A curated set of 139 metabolic enzymes was compared against 500 random protein sets of the same size and against random sets additionally matched on abundance, missingness, variance and co-regulation. Classification reached a macro-average AUROC of 0.901 (95% CI 0.871–0.933) under cross-validation and 0.862 in the untouched expansion cohort; methylation subtypes were classified with comparable accuracy (0.905). Subtype proportions in TCGA-KIRC differed from CPTAC by at most 2.5 percentage points. Among 518 patients with complete clinical data, grade improved a model containing age and stage (*p* = 0.001), whereas subtype added nothing once grade was included (*p* = 0.703; change in C-index +0.0019, 95% CI −0.0023 to +0.0116); with 171 deaths, the study could detect a hazard ratio of 1.64 at 80% power, so small effects remain possible. The metabolic panel did not outperform size-matched (*p* = 0.649) or covariate-matched random sets (*p* = 0.762), whereas a housekeeping set performed significantly worse. CPTAC consensus subtype identity is highly predictable from the proteome but redundantly encoded rather than concentrated in a functional class, and adds no measurable prognostic information beyond stage and grade in this cohort. High classification accuracy for a molecular label is not evidence of clinical utility; these conclusions apply to the CPTAC NMF scheme as evaluated here and should not be extended to proteomic subtyping in general.

## 1. Introduction

Clear cell renal cell carcinoma accounts for roughly three quarters of renal cell carcinomas and for most deaths from the disease. Its defining molecular event is biallelic loss of the von Hippel–Lindau tumor suppressor, which occurs in the large majority of sporadic cases and removes the mechanism by which hypoxia-inducible factor alpha subunits are degraded under normoxia [1]. The resulting constitutive HIF activity, dominated by HIF-2 alpha in this tumor, drives a transcriptional program that would normally be reserved for hypoxic conditions [2].

The metabolic consequences are consistent enough that ccRCC is often described as a metabolic disease as much as a genetic one [3]. Glycolytic enzymes and glucose transporters rise while oxidative phosphorylation and fatty acid oxidation fall, and neutral lipid accumulates in the cytoplasm to produce the clear cell appearance the tumor is named after [4]. Genomic characterization of the disease placed these changes alongside recurrent alterations in chromatin regulators and located them within a broader shift in nutrient handling [5]. Recent work has extended this to individual enzymes that may be actionable, including nicotinamide N-methyltransferase, which is markedly overexpressed in tumor tissue and has been proposed as a metabolic drug target [6]. The same program has also been exploited therapeutically: allosteric inhibition of HIF-2 alpha showed activity in von Hippel–Lindau disease [7] and in sporadic advanced ccRCC in early-phase testing [8], and belzutifan subsequently improved progression-free survival compared with everolimus in a randomized trial [9]. The metabolic phenotype of ccRCC thus has therapeutic as well as descriptive relevance.

Molecular subtypes have been described against this shared background for more than a decade. A meta-analysis of transcriptomic datasets defined the ccA and ccB groups and showed that they separate on survival [10]. A 34-gene classifier derived from that work, ClearCode34, was developed as a prognostic tool for localized disease [11] and later reviewed across independent cohorts [12]. The scheme was extended to metastatic disease, where subtype assignment varied within tumors and related to aggressiveness [13], and was tested for whether it adds to the established International Metastatic RCC Database Consortium risk model [14]. Whether a molecular subtype improves on clinical variables already in use has thus been an explicit question in this field for some time.

Proteogenomic characterization brought a different set of measurements to the same problem. The CPTAC discovery cohort of 103 tumors integrated genomic, transcriptomic, proteomic and phosphoproteomic data and reported consensus subtypes together with immune and methylation classifications [15]. A confirmatory cohort expanded this to 213 cases with intratumoral sampling and histopathological annotation, and it is the source of the subtype labels used here [16]. Pan-cancer resources assembled from these projects have made the underlying matrices broadly reusable [17], and comparable analyses across tumor types have been used to nominate therapeutic targets [18].

Two questions about such subtypes are not resolved by the studies that define them. The first concerns information content. A label built by integrating several molecular layers need not be recoverable from any single layer, and where that information sits in the proteome is not determined by how the label was constructed. This is relevant because feature sets selected on biological grounds are routinely presented as evidence that particular proteins or genes are important. The ccRCC literature contains many transcriptomic risk signatures built on ferroptosis-related long non-coding RNAs [19], m6A methylation regulators [20], ferroptosis-related genes [21] and metabolic gene sets [22]. These studies report survival separation for the selected panel, but a comparison against randomly chosen panels of equal size is not usually presented, and without it a good classification result does not distinguish a biologically privileged set from an arbitrary one of the same dimension.

The second question concerns clinical value. Subtype labels derived within a single cohort may end up describing that cohort instead of the disease, and the corresponding check, applying the classifier to an independent population profiled on a different platform, is often omitted. Where prognostic claims are made, the appropriate comparison is against stage and grade rather than against an unadjusted model, since validated tools such as the SSIGN score already combine those variables and perform well [23].

We addressed both questions in the CPTAC ccRCC confirmatory cohort. Classifiers for the consensus subtypes were trained on the proteome under nested cross-validation, and separately under a locked hold-out design in which model development was confined to one half of the cohort. Because the consensus labels were derived from data that included the proteome, the analysis was repeated with methylation subtypes, which were defined without proteomic input. A transcriptome version of the classifier was transferred to TCGA-KIRC [24], and the transferred subtypes were tested for incremental prognostic value against age, stage and Fuhrman grade. Finally, the curated metabolic feature set was compared against random sets of the same size.

## 2. Materials and Methods

### 2.1. Data Sources

Processed DIA proteomics for the CPTAC ccRCC confirmatory cohort [16] were obtained from the Proteomic Data Commons (study PDC000411, publication supplementary archive for PMID 36563681). Clinical annotation, NMF consensus subtypes, methylation and immune subtypes, mutation status and outcome data came from Supplementary Table S7 of the source publication [16]. Transcriptomic data were obtained through the cptac Python package. Targeted metabolomics covering 183 metabolites in 50 tumors and 7 normal specimens came from Supplementary Table S1 of the same source. TCGA-KIRC [24] STAR-TPM expression, phenotype and survival tables were downloaded from UCSC Xena [25], with Ensembl identifiers mapped to symbols using the accompanying gencode v36 probemap. CRISPR gene effect scores (Chronos [26]) and cell line annotation came from DepMap. Guide-level mis-annotation in pooled CRISPR libraries is a recognized source of bias in such screens [27].

### 2.2. Proteomic Preprocessing

Intensities were log2 transformed and each sample aligned to the cohort median. Samples with more than 50 percent missing values were removed, as were cases absent from the clinical table. Proteins quantified in fewer than 70 percent of tumors were dropped. Missing values were imputed by the median within cross-validation folds, never on the full matrix [28,29]. Missing value patterns and cohort structure in principal component space are shown in Figure S2.

### 2.3. Pathway Scoring

Tumor against matched normal comparison used a paired *t*-test on log2 intensities across the 166 patients with both specimens, with Benjamini–Hochberg correction across proteins; effect sizes are mean paired differences on the log2 scale. Proteins detected in one tissue type but not the other were analyzed separately by detection frequency, since paired testing is undefined for them. Twenty-two metabolic pathways were curated from KEGG and Reactome, covering 273 gene symbols. Pathway scores are the mean z-score of member proteins. Protein-level and pathway-level comparisons are separate calculations: protein-level effects are mean paired log2 differences, while pathway-level effects are reported as Cohen’s d on the pathway score, since scores are already standardized and a raw difference would not be interpretable. For tumor against normal comparison, z-scoring used tumor and normal samples together so that the group difference is preserved; scoring each matrix separately centers both at zero and removes the effect being measured. Pathways with fewer than three quantified members were dropped.

### 2.4. Classification

Features were reduced to the 3000 most variable proteins using an unsupervised variance filter. Five algorithms were compared: L2-penalized logistic regression, linear discriminant analysis, random forest, extra trees and RBF-kernel SVM. The outer loop was 5-fold stratified cross-validation with 2 repeats; the inner loop was 3-fold and tuned the number of selected features alongside algorithm-specific hyperparameters. Univariate selection retained at most 150 proteins, so a candidate set smaller than this threshold entered the classifier in full while the 3000-protein pool was reduced to 150; this asymmetry favors the larger pool and therefore does not inflate the curated set. Imputation, standardization and univariate feature selection were fitted inside each training fold, never on the full matrix, because missingness in data-independent acquisition proteomics is not random and imputation strategy affects downstream inference [28,29,30]. Performance is reported as macro one-versus-rest AUROC with percentile bootstrap confidence intervals over 300 resamples, balanced accuracy, per-class AUROC and per-class Brier score.

### 2.5. Locked Hold-Out Design

Model development, univariate feature selection and hyperparameter tuning were confined to one cohort using three-fold internal cross-validation. The resulting estimator was locked and applied once to the other cohort, which was untouched during development. Both directions were run. Reported metrics are macro one-versus-rest AUROC with percentile bootstrap intervals over 500 resamples, balanced accuracy, per-class AUROC, sensitivity, precision, Brier score and the confusion matrix.

### 2.6. Classification of Methylation Subtypes

Methylation subtype labels (Methyl1 to Methyl3) were taken from the same clinical table. These were derived from methylation data without proteomic input. The classification pipeline was identical to that used for the NMF labels.

### 2.7. Null Distribution for Feature Sets

The metabolic subset comparison used identical cross-validation splits. To build a null distribution, 500 subsets of the same size were drawn without replacement from the 3000-protein pool and evaluated under the same splits. The housekeeping negative control was compared against its own size-matched null of 500 draws of 92 proteins. Hyperparameters were fixed (L2 logistic regression, C = 0.5, 150 selected features) rather than tuned, since tuning inside every draw was not computationally practical; the same fixed settings were applied to the metabolic subset, the housekeeping subset and the full pool for comparability. Because the selection cap of 150 features exceeded the size of the 139-protein and 92-protein sets, no selection occurred within those sets and all their features entered the model; within the 3000-protein pool, 150 features were selected. The empirical *p* value is the proportion of random subsets scoring at or above the metabolic subset, with a plus-one correction. A feature-count curve was constructed by drawing 30 random subsets at each of eight sizes between 10 and 3000, summarized by median and 2.5th to 97.5th percentile. A negative-control subset was formed from proteins whose symbols begin with ribosomal, spliceosomal or proteasomal prefixes.

### 2.8. SHAP Analysis

SHAP values were computed from the best model refitted on the full analysis cohort. These describe the fitted model and are not a performance estimate. To test whether the ranking was driven by measurement depth rather than biology, SHAP importance was correlated against mean protein abundance (Spearman rho = −0.04, *p* = 0.61; Figure S1).

### 2.9. Cross-Platform Transfer

A transcriptome classifier was trained on the 103 CPTAC cases with both RNA data and a subtype label, using the same pipeline. CPTAC and TCGA matrices were restricted to shared gene symbols and each gene was z-scored within its cohort. Only primary tumor specimens (sample code 01) were retained from TCGA, with replicate samples averaged per patient.

### 2.10. Survival Analysis

The prespecified analysis was a three-group log-rank test on predicted subtype. Pairwise comparisons, the NMF3 against remainder contrast, landmark analyses and restricted mean survival time were chosen after inspecting the Kaplan–Meier curves and are labeled exploratory throughout. Incremental prognostic value was assessed by comparing nested Cox models with a likelihood-ratio test and by the change in Harrell’s C-index. Subtype entered as a three-level categorical variable with NMF1 as reference, so the likelihood-ratio test carries two degrees of freedom. A binary NMF3-against-remainder contrast was retained as a secondary exploratory analysis. Cox models included age and stage collapsed to I–II against III–IV, with and without predicted subtype. Proportional hazards were assessed with Schoenfeld residuals under rank transformation. Because violations were detected, follow-up was additionally split into non-overlapping windows at two and four years and restricted mean survival time was computed to five years with a bootstrap interval over 500 resamples. To test whether prediction error attenuated the association, the Cox model was refitted in subsets defined by assignment probability thresholds of 0.8 and 0.9. Fuhrman grade was taken from the legacy TCGA clinical matrix, since the field is empty in the GDC-harmonized release, and dichotomized as G1–G2 against G3–G4 with GX excluded. All models in a sequence were restricted to patients with complete data for every covariate in the largest model. A sensitivity analysis stratified the baseline hazard by the four stage groups.

### 2.11. Software

Analyses used Python 3.11.9 with scikit-learn 1.5.2 [31], pandas 2.2.3, numpy 1.26.4, scipy 1.13.1, statsmodels 0.14.4, shap 0.46.0 [32] and lifelines 0.30.0 [33]. Reporting follows TRIPOD+AI [34] where applicable. The complete analysis pipeline, including the scripts that generated every figure and table and the random seeds needed to reproduce the reported values, is archived on Zenodo (https://doi.org/10.5281/zenodo.22660275).

## 3. Results

### 3.1. Cohort and Data

The CPTAC ccRCC confirmatory cohort [16] contains 213 cases, split between an initial set of 103 and an expansion set of 110. Data-independent acquisition proteomics covered 222 tumor and 171 adjacent normal specimens. One tumor sample with more than half its values missing was removed. After log2 transformation, sample-wise median alignment and a requirement that a protein be quantified in at least 70 percent of samples, 5771 proteins remained, with 4.8 percent missing values.

The analysis cohort was the 211 tumors carrying an NMF consensus subtype label. Cohort characteristics are given in Table 1.

Principal component analysis separated the initial and expansion sets along the third component (Cohen’s d = −1.32). At the pathway level, 11 of 22 metabolic pathway scores differed between the two sets, and all 11 differences pointed the same way. A uniform shift of this kind is a technical signature rather than a biological one. We did not apply batch correction, which would have risked removing real variation along with it, and we did not supply cohort membership to the classifier as a feature. The locked hold-out analysis in Section 3.3 consequently carries whatever batch structure exists between the two sets, and we return to this in the limitations.

### 3.2. Subtype Classification from the Proteome

Before modeling we reduced the feature space to the 3000 most variable proteins. The filter used no label information, so it does not leak outcome into the cross-validation.

Five algorithms were compared under 5-fold, 2-repeat nested cross-validation with imputation, scaling, feature selection and hyperparameter tuning all performed inside the resampling loop. Results are given in Table 2. L2-penalized logistic regression performed best, with a macro AUROC of 0.901 (0.871 to 0.933) and balanced accuracy of 0.754. The other four fell within a narrow band.

**Figure 1:**
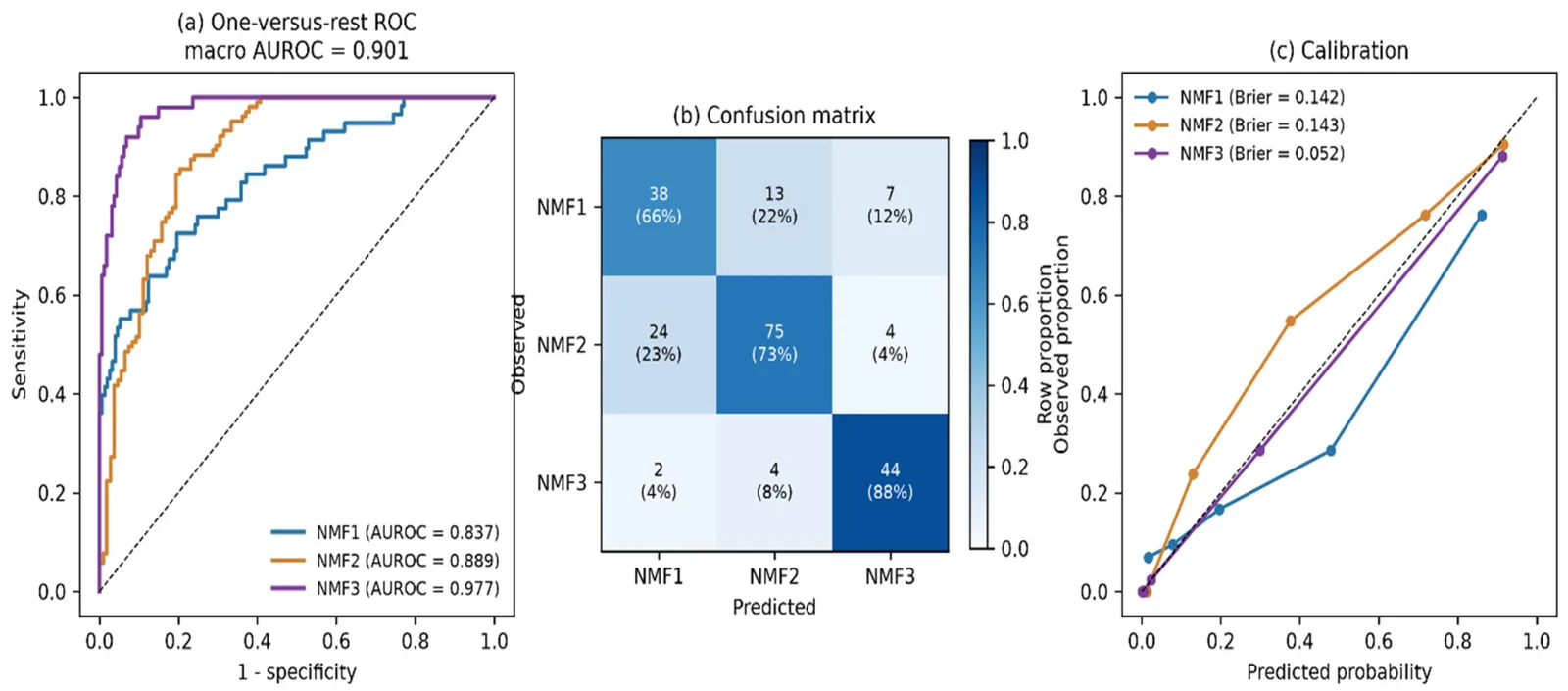
Subtype classification from the proteome. (**a**) One-versus-rest ROC curves for the L2-penalized logistic regression model under nested cross-validation. (**b**) Confusion matrix with row percentages. (**c**) Calibration curves and Brier scores by class. Dashed lines mark chance performance in (**a**) and perfect calibration in (**c**).

Performance was uneven across the three classes (Figure 1). NMF3 was recovered cleanly (AUROC 0.977, sensitivity 88 percent, Brier score 0.052) and its calibration curve tracked the diagonal. NMF1 and NMF2 were harder to separate from each other: 24 of 103 NMF2 tumors were assigned to NMF1 and 13 of 58 NMF1 tumors to NMF2, while confusion between either and NMF3 was rare. Brier scores were 0.142 for NMF1 and 0.143 for NMF2 against 0.052 for NMF3.

### 3.3. Locked Hold-Out Validation

Cross-validated estimates share information across folds. To provide a test on untouched samples with observed labels, we used the existing cohort structure. Model development, feature selection and hyperparameter tuning were confined to the CPTAC initial cohort (*n* = 103), the resulting model was locked, and it was applied once to the expansion cohort (*n* = 108). The reverse direction was run as a sensitivity analysis.

**Figure 2:**
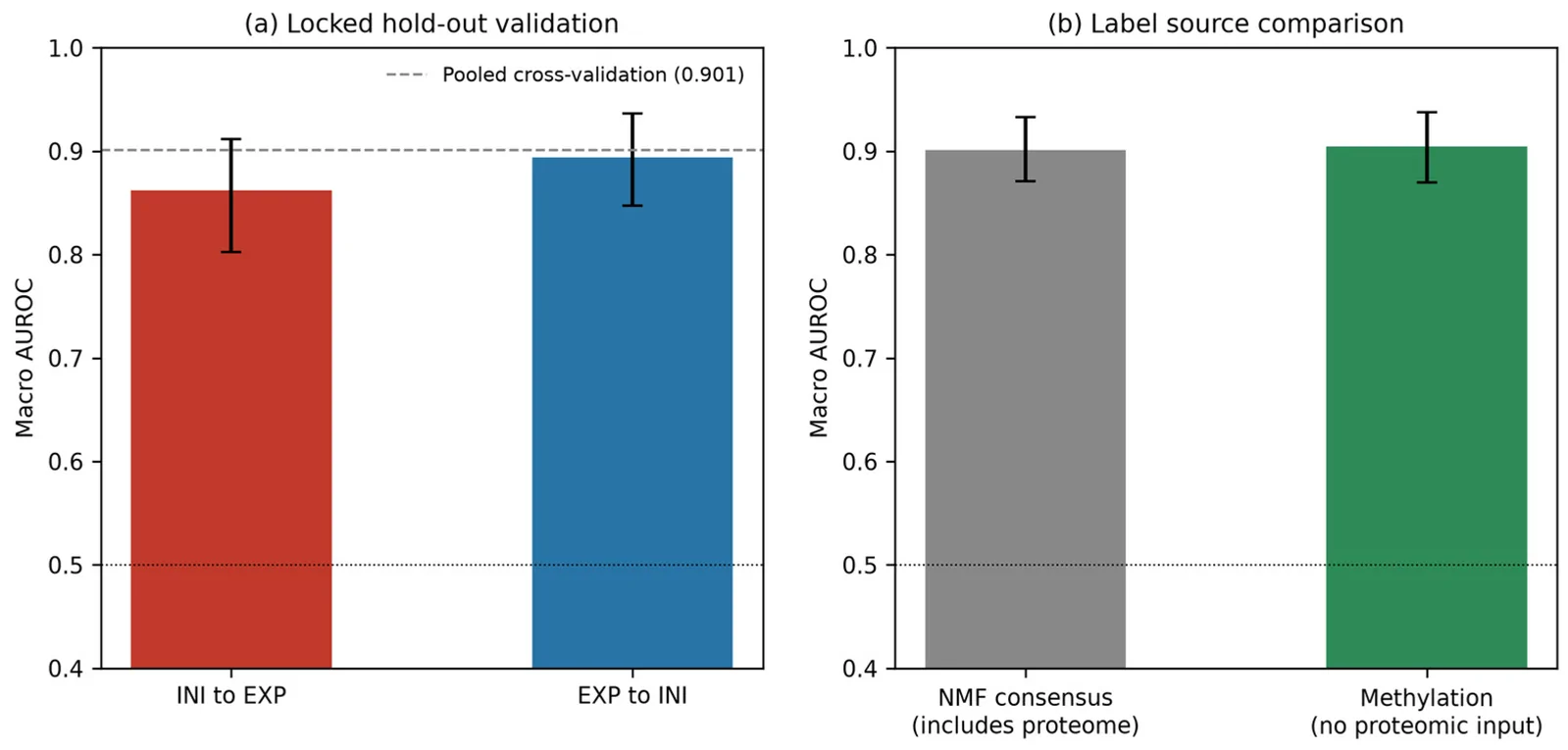
Locked hold-out validation and label-source comparison. (**a**) Macro AUROC for both hold-out directions against the pooled cross-validated value. (**b**) NMF subtype classification against methylation subtype classification. Error bars are 95% bootstrap intervals; the dashed line marks the pooled cross-validated value (0.901) and the dotted lines chance performance (0.5). INI, initial cohort; EXP, expansion cohort.

Results are given in Table 3. Training on the initial cohort gave a macro AUROC of 0.862 (0.802 to 0.914) in the expansion cohort with balanced accuracy of 0.700. Training on the expansion cohort gave 0.894 (0.848 to 0.937) in the initial cohort with balanced accuracy of 0.769 (Figure 2a). The class pattern seen under cross-validation persisted in both directions: NMF3 was recovered almost perfectly (AUROC 0.959 and 0.988, Brier 0.065 and 0.039), while NMF1 was recognized poorly (sensitivity 0.42 and 0.56).

Both hold-out estimates fall below the pooled cross-validated value of 0.901. Two explanations are available and this design cannot separate them: the development sets contain only about 100 tumors each, and the two cohorts differ along the third principal component (Section 3.1). We report the hold-out figures as the more conservative estimate.

### 3.4. Classification of a Label Defined Without Proteomic Input

The NMF consensus labels were derived from multi-omic data that included the proteome, so predicting them from the proteome is partly circular. The same cohort carries methylation subtypes (Methyl1, Methyl2, Methyl3), which were defined from methylation data alone and are therefore independent of the proteome. Applying the same pipeline to the 211 tumors with methylation labels (59, 77 and 75 per class) gave a macro AUROC of 0.905 (0.870 to 0.938) with balanced accuracy of 0.777 (Figure 2b). Per-class values were 0.939 for Methyl1, 0.851 for Methyl2 and 0.924 for Methyl3. Proteomic data classify a label that made no contribution to its own construction as accurately as they classify the NMF labels. This does not eliminate the circularity concern for the NMF result, but it shows that the performance level observed here is achievable without it. Two distinct questions should be separated. Whether the proteome carries subtype-relevant biology is addressed by the methylation result, and the answer is affirmative. Whether the specific figure of 0.901 for the NMF labels is inflated by shared construction is not addressed by it, because both labels may be driven by the same underlying biology, and we cannot partition the NMF estimate into circular and non-circular components.

### 3.5. Feature Sets Against a Null Distribution

Because ccRCC is metabolically distinctive, we asked whether a curated set of metabolic enzymes carries the subtype signal preferentially. Of the 273 gene symbols in our 22 pathway definitions, 139 were present in the analysis matrix. Under identical cross-validation splits and the same algorithm, this subset gave a macro AUROC of 0.905, against 0.901 for the full 3000-protein pool (Table 2). Considered on its own, this comparison would suggest that subtype identity is concentrated in metabolism.

**Figure 3:**
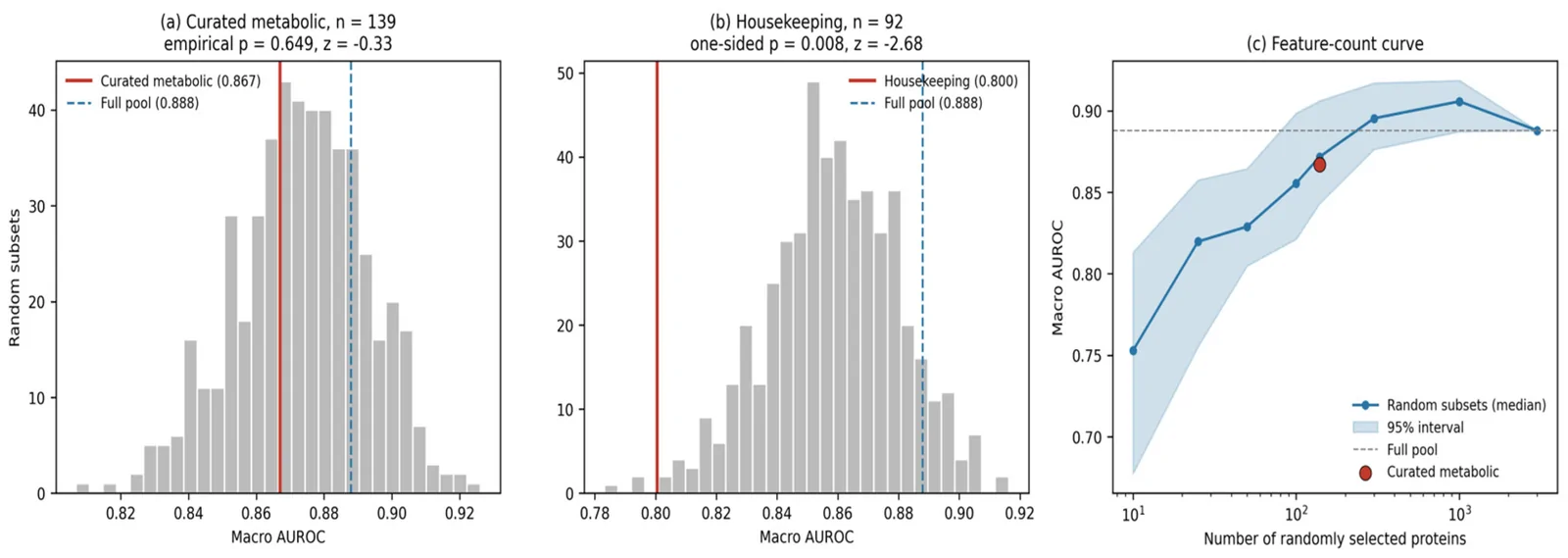
Null distribution for feature sets. (**a**) Macro AUROC for 500 random subsets of 139 proteins, with the curated metabolic subset and the full pool marked. (**b**) The same comparison for 500 size-matched subsets of 92 proteins against the housekeeping subset. (**c**) Feature-count curve from 30 random draws at each size, shown as median and 95% interval.

This apparent advantage does not hold against an empirical null distribution. We drew 500 random subsets of 139 proteins from the same pool and evaluated each under the same splits, holding hyperparameters fixed for computational tractability across the repeated refits. Under fixed hyperparameters the full pool scored 0.888 and the metabolic subset 0.867. The random subsets averaged 0.874 with a standard deviation of 0.019 and a range of 0.807 to 0.926. The empirical *p* value was 0.649 and the z score −0.33 (Figure 3a). Repeating the same comparison with an independent set of draws gave 0.651 and −0.30, so the estimate is stable to the random draw.

A feature-count curve indicates why. Using 30 random draws at each size, median macro AUROC rose from 0.753 at 10 proteins to 0.820 at 25, 0.856 at 100, 0.872 at 139, 0.895 at 300 and 0.906 at 1000 (Figure 3c). Much of the available subtype information was recovered by relatively small random subsets, with progressively smaller gains beyond roughly 100 to 300 proteins. Performance does not plateau exactly at 139 proteins, but the gradient in that region is shallow enough that any set of 139 proteins drawn from this matrix lands in the same neighborhood.

Saturation does not imply that feature identity is irrelevant. A size-matched comparison for 92 ribosomal, spliceosomal and proteasomal proteins placed that set at 0.801 against a random mean of 0.859 for 500 subsets of the same size, a z score of −2.68 and a one-sided *p* of 0.008 (Figure 3b). Housekeeping machinery carries measurably less subtype information than an arbitrary set of proteins does, while metabolic enzymes carry an ordinary amount. Subtype information is spread widely across the proteome, but it is not spread uniformly.

To test whether the curated set performed as it did because of how well its proteins are measured rather than what they do, random panels were additionally matched to it on mean log2 abundance, fraction missing, variance and mean absolute correlation with the pool. Matching was cumulative over four levels. For each protein in the curated set the 25 nearest pool proteins in standardized covariate space were identified, excluding members of the curated set, and one was drawn at random without replacement within a panel; 100 panels were drawn per level. Balance was assessed as the standardized mean difference between the curated set and the matched draws, scaled by the protein-level standard deviation in the pool. The same procedure was applied to the housekeeping set.

**Figure 4:**
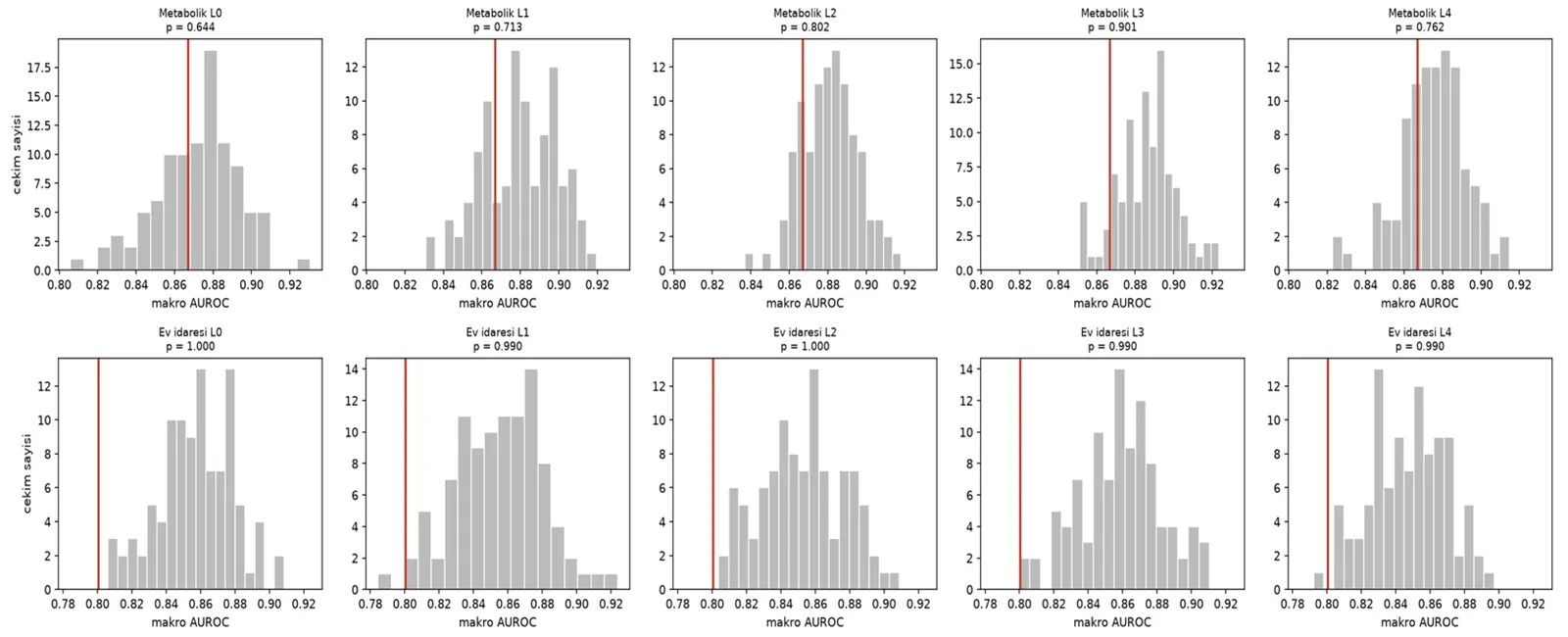
Covariate-matched null distributions. Macro AUROC for 100 random panels drawn at each matching level, for the curated metabolic set ((**top**) row, 139 proteins) and the housekeeping set ((**bottom**) row, 92 proteins). L0 matches size only; L1 adds mean abundance; L2 adds missingness; L3 adds variance; L4 adds mean absolute correlation with the pool. The vertical line marks the observed value.

Metabolic enzymes are not a random sample of the proteome with respect to measurement properties. Relative to the 3000-protein pool, the curated set was more abundant (+0.76 SD), less often missing (−0.44 SD), slightly more variable (+0.15 SD) and more strongly co-regulated with the rest of the pool (+0.65 SD). A size-matched random draw does not control for the possibility that the curated set classifies well simply because it is better measured. We repeated the comparison against random panels matched to the curated set on these characteristics in cumulative steps, holding the splits, the algorithm and the hyperparameters fixed. Matching moved the null distribution upward rather than downward: mean macro AUROC rose from 0.872 for size-matched draws to 0.879 with abundance matched, 0.881 with missingness added, 0.886 with variance added, and 0.875 in the fully matched comparison. The curated set stayed at or below the null mean at every level, with empirical *p* values of 0.644, 0.713, 0.802, 0.901 and 0.762 and a z score of −0.48 when all four characteristics were matched (Figure 4). Covariate balance in the fully matched draws was within 0.07 SD on every characteristic.

The housekeeping comparison ran in the opposite direction and was equally stable. Against fully matched draws the 92 ribosomal, spliceosomal and proteasomal proteins scored 0.801 against a null mean of 0.847, a z score of −2.09, and the deficit held at every matching level (z from −2.62 to −2.09). The matched null can separate a genuinely un-informative feature class from an ordinary one, which is what makes the flat result for metabolic enzymes interpretable instead of merely underpowered.

### 3.6. Cross-Platform Transfer to TCGA-KIRC

TCGA-KIRC does not carry CPTAC consensus subtype labels, so applying the classifier there cannot estimate classification accuracy. What it can test is whether the subtype structure appears in an independent population profiled on a different platform. We describe this as cross-platform transfer rather than external validation.

To test the subtypes outside CPTAC we trained a transcriptome classifier on the 103 CPTAC cases with RNA data (internal macro AUROC 0.847) and applied it to 533 primary TCGA-KIRC tumors. Ensembl identifiers were mapped to symbols and 57,231 genes were shared. Each gene was z-scored within its own cohort to remove platform offset.

**Figure 5:**
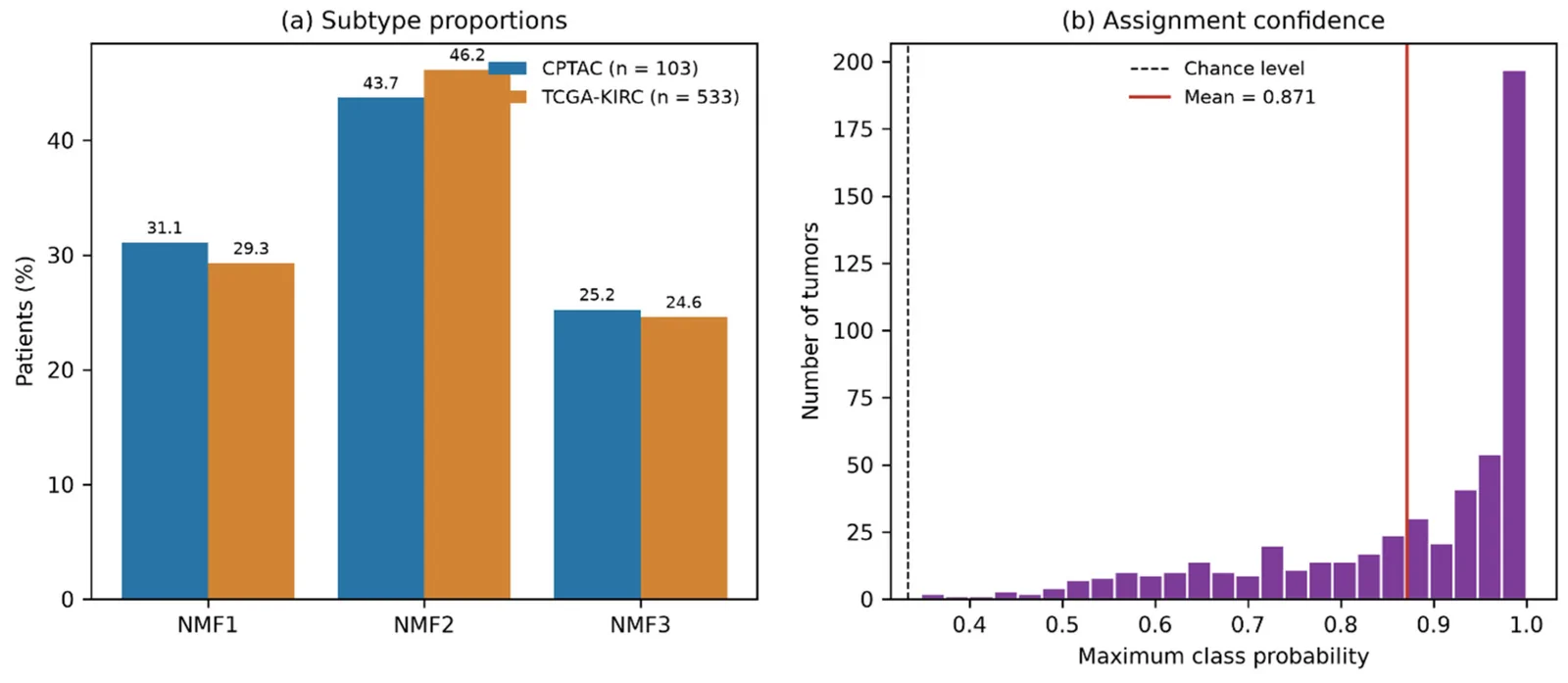
Transfer to TCGA-KIRC. (**a**) Predicted subtype proportions in 533 TCGA-KIRC tumors against observed proportions in CPTAC. (**b**) Distribution of maximum class probability.

Predicted subtype proportions in TCGA-KIRC were 29.3, 46.2 and 24.6 percent for NMF1, NMF2 and NMF3, against 31.1, 43.7 and 25.2 percent in the 103 CPTAC cases with transcriptomic data that trained the classifier (Figure 5). These differ from the proportions in Table 1, which describes all 211 tumors in the analysis cohort. Mean assignment probability was 0.871, and most tumors were assigned with probability above 0.9. Reproducing the class balance to within 2.5 percentage points, across a different platform and a cohort five times larger, is consistent with the classifier tracking a structure present in the disease rather than only in the training cohort. Agreement of marginal proportions is nonetheless a weak criterion: it is compatible with substantial per-patient misassignment, and without ground-truth labels in TCGA-KIRC the accuracy of individual assignments there cannot be estimated. The prognostic analyses that follow rest on predicted labels and inherit this limitation, which Section 3.8 addresses by repeating them with continuous class probabilities.

### 3.7. Incremental Prognostic Value Against Stage and Grade

**Figure 6:**
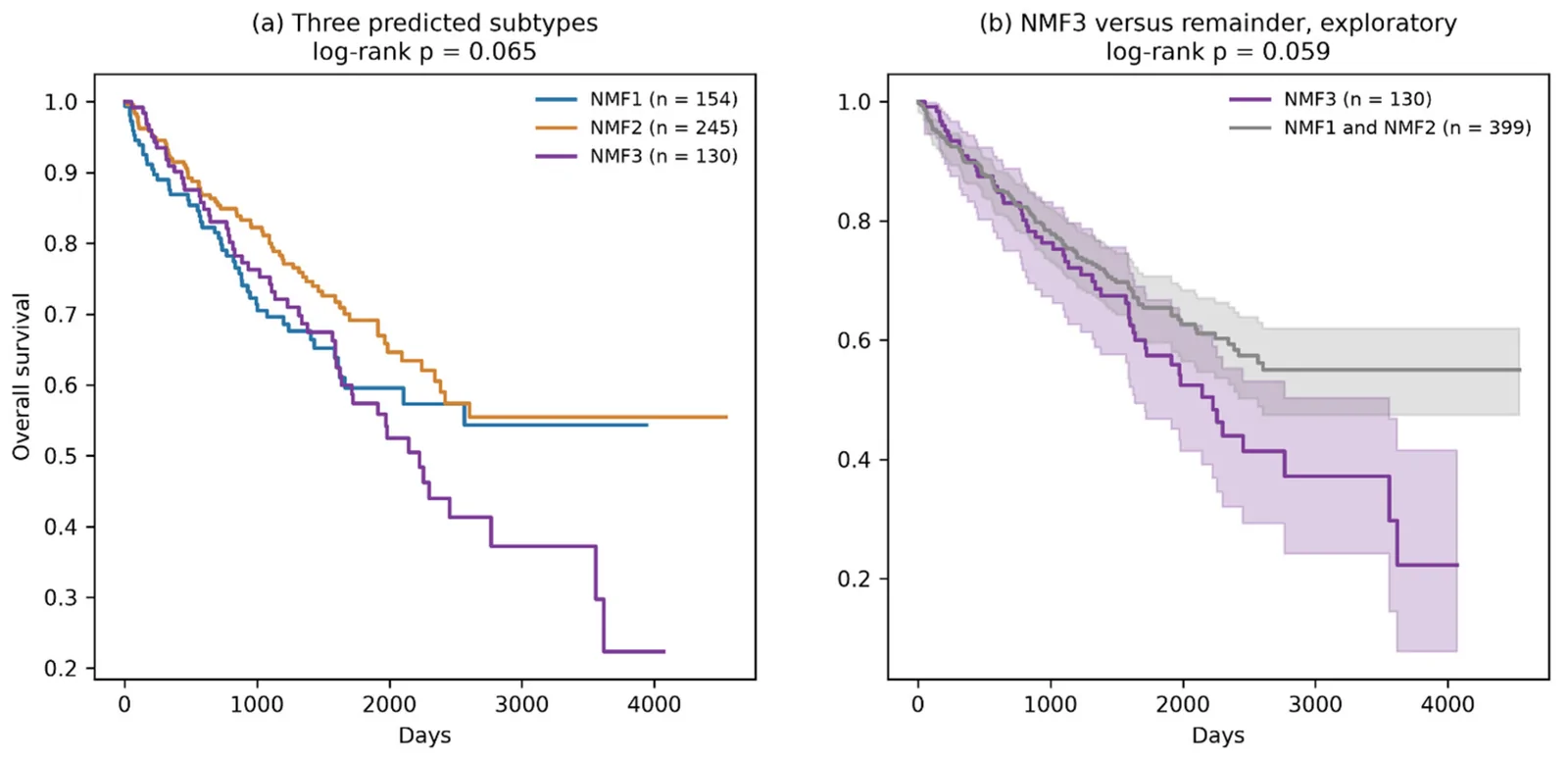
Overall survival in TCGA-KIRC by predicted subtype. (**a**) Three-group comparison, prespecified. (**b**) NMF3 against the remaining tumors, exploratory.

Survival data were available for 529 TCGA-KIRC tumors, with 173 deaths and a median follow-up of 1191 days. The prespecified three-group log-rank test gave *p* = 0.065. Kaplan–Meier curves for NMF1 and NMF2 overlapped, while NMF3 separated after roughly 1600 days (Figure 6).

Rather than relying on a single hazard ratio, we compared nested models directly, entering subtype as a three-level categorical variable with NMF1 as reference. In the 526 patients with age and stage recorded, a model containing those two variables gave a C-index of 0.7356; adding subtype moved it to 0.7389, with a likelihood-ratio chi-square of 2.318 on two degrees of freedom, *p* = 0.314. Neither contrast approached significance: 0.80 (0.56 to 1.16) for NMF2 against NMF1 and 1.03 (0.70 to 1.52) for NMF3 against NMF1, while stage III-IV carried a hazard ratio of 3.75. Table 4 reports the full model sequence on the smaller set of patients for whom grade was also available, so that grade and subtype can be compared as increments in identical samples.

**Figure 7:**
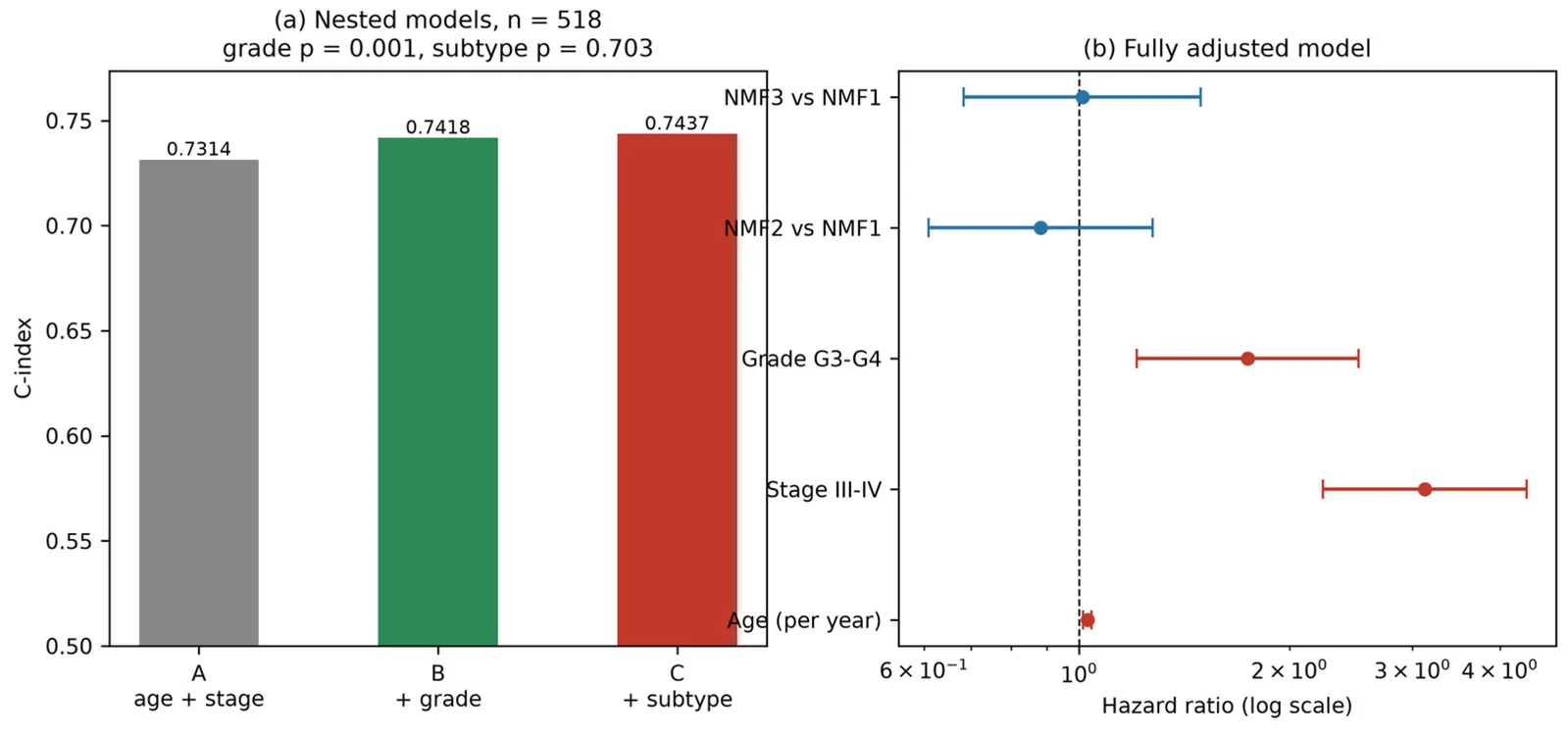
Incremental prognostic value with subtype as a three-level categorical variable. (**a**) Three nested models: age and stage, with grade added, and with grade and subtype. (**b**) Hazard ratios with 95% intervals from the multivariable Cox model, NMF1 as reference; the dashed line marks a hazard ratio of 1.

The three models were fitted on identical patients so that the two increments can be compared directly. Fuhrman grade, taken from the legacy TCGA release because the field is empty after GDC harmonization, was available for 518 patients, 279 of them (54 percent) with G3 or G4 disease. Adding grade to age and stage improved the model (*p* = 0.001, C-index +0.0103). Adding subtype on top of grade did not (*p* = 0.703, C-index +0.0019) (Figure 7a). The same model structure, in the same patients, detects an established prognostic variable and does not detect subtype (Figure 7b).

Because stage violated the proportional hazards assumption, we repeated the analysis with stage as a stratification factor, allowing the baseline hazard to differ across the four stage groups. Subtype remained without incremental value (chi-square 0.700, df 2, *p* = 0.705), while grade retained its association (HR 1.55, 1.06 to 2.26, *p* = 0.022). The null result for subtype is therefore not an artifact of the proportional hazards assumption for stage.

A secondary contrast of NMF3 against the remaining tumors, chosen after inspecting the survival curves and reported as exploratory, gave a hazard ratio of 1.18 (0.85 to 1.63, *p* = 0.33) with a likelihood-ratio *p* of 0.333 on one degree of freedom. Grouping NMF1 with NMF2 raises the apparent NMF3 effect because NMF1 itself carried an intermediate event rate, which is one reason the three-level analysis is the appropriate primary test.

Because TCGA subtypes are predicted rather than observed, misclassification could attenuate a real association toward the null. If that were operating, restricting the analysis to confidently assigned tumors should move the hazard ratio away from unity. It moved toward unity instead: for the NMF3 contrast, 1.18 in all 526 patients, 1.10 in the 387 with assignment probability of at least 0.8, and 0.98 in the 306 with at least 0.9. These findings provide no evidence that low-confidence assignment materially attenuated the observed association, though confidence-based subsetting is itself a form of selection and does not eliminate misclassification.

Proportional hazards testing on Schoenfeld residuals flagged violations for stage (*p* < 0.0001) and for the binary subtype contrast (*p* = 0.0078). Splitting follow-up into non-overlapping windows located the apparent subtype effect entirely in the late period (Figure S3): NMF3 against the others gave *p* = 0.947 in the first two years, *p* = 0.372 in years two to four, and *p* = 0.003 beyond year four. These window analyses are unadjusted for stage and are reported as exploratory. Restricted mean survival time over five years differed by 33 days between NMF3 and the others, with a bootstrap interval of −97 to 144 days.

### 3.8. Sensitivity Analyses

Several choices in the primary analysis could in principle have produced the negative prognostic result or the flat null comparison. Each was varied in turn, holding everything else fixed.

**Figure 8:**
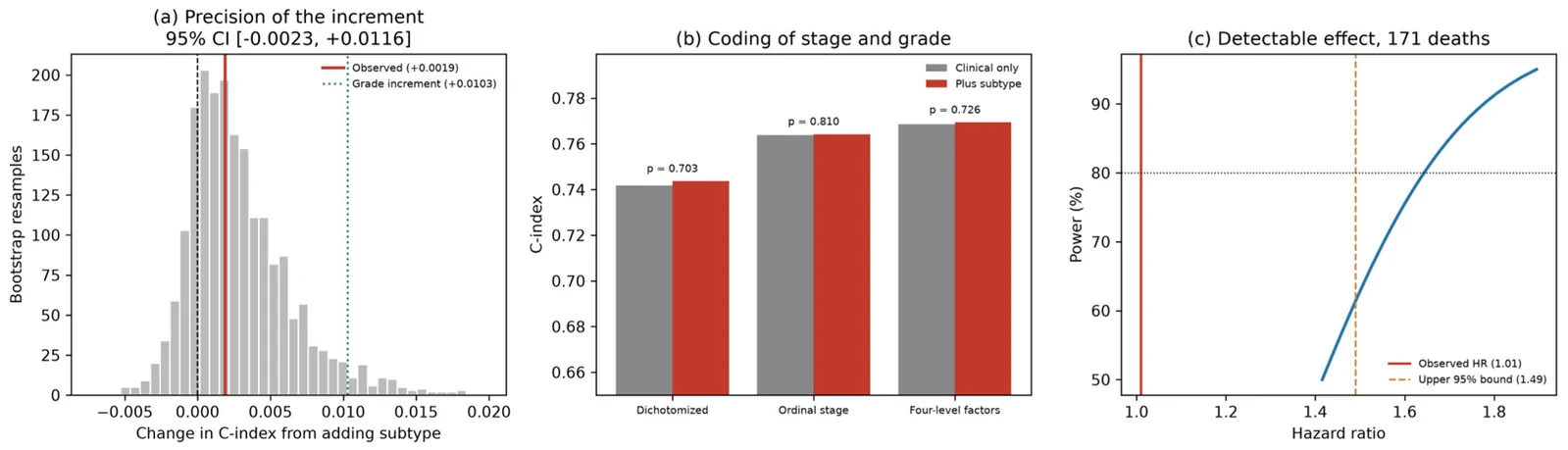
Sensitivity analyses for the prognostic comparison. (**a**) Bootstrap distribution of the change in C-index from adding subtype, with the increment contributed by grade marked for reference. (**b**) C-index for the clinical model and the clinical model plus subtype under three codings of stage and grade. (**c**) Detectable hazard ratio as a function of power, given 171 deaths. In (**a**), the dashed line marks zero change, the solid line the observed increment and the dotted line the increment contributed by grade; in (**c**), the solid vertical line marks the observed hazard ratio, the dashed vertical line its upper 95% confidence bound and the dotted horizontal line 80% power.

Precision of the increment. A non-significant likelihood-ratio test does not establish equivalence, so the change in C-index from adding subtype was bootstrapped over 2000 resamples. The observed increment was +0.0019 with a 95 percent interval of −0.0023 to +0.0116, and 81.5 percent of resamples showed some improvement (Figure 8a). The data therefore exclude a gain larger than about 0.012 C-index units. Adding grade to age and stage in the same patients gained 0.0103, which lies inside that interval, so an improvement of the size contributed by grade cannot be formally excluded even though the point estimate for subtype is roughly one fifth of it.

Statistical power. With 171 deaths and the observed NMF3 proportion of 0.247, the study could detect a hazard ratio of 1.64 or larger at 80 percent power and 1.77 at 90 percent power for a single binary contrast (Figure 8c). The observed hazard ratio was 1.01 with an upper confidence bound of 1.49, both below the detectable range. The result is the absence of a moderate-to-large independent effect rather than evidence that no effect exists.

Probabilistic rather than hard class assignment. Entering the three continuous class probabilities directly, with no thresholding and no confidence-based subsetting, gave a C-index of 0.7433 against 0.7418 for the clinical model, with a likelihood-ratio chi-square of 0.516 on two degrees of freedom, *p* = 0.773. Hazard ratios per unit probability were 0.90 (0.58 to 1.40) for NMF2 and 1.05 (0.67 to 1.65) for NMF3, while grade retained a hazard ratio of 1.74 (1.21 to 2.51, *p* = 0.003) in the same model. Discretizing the classifier output did not create the null result.

Coding of stage and grade. Dichotomization discards information, so the nested comparison was repeated with both variables as ordinal scores, as four-level factors, and within a stage-and-grade composite. Richer coding improved the clinical model substantially, raising the C-index from 0.7418 to 0.7638 for ordinal coding and 0.7686 for four-level factors, which confirms that dichotomization costs discrimination. The subtype increment nonetheless remained absent and became smaller rather than larger, with likelihood-ratio *p* values of 0.703, 0.810, 0.726 and 0.716 and increments of +0.0019, +0.0004, +0.0008 and +0.0009 (Table 5, Figure 8b).

Time-varying subtype effect. Rather than selecting follow-up windows after inspecting the curves, subtype was given a coefficient varying with log time in a counting-process model with 1950 episodes. Neither interaction term approached significance, and the joint test for a time-varying subtype effect gave chi-square 1.878 on two degrees of freedom, *p* = 0.391. The apparent late separation reported in Section 3.7 is therefore best regarded as a consequence of post-hoc window selection.

**Figure 9:**
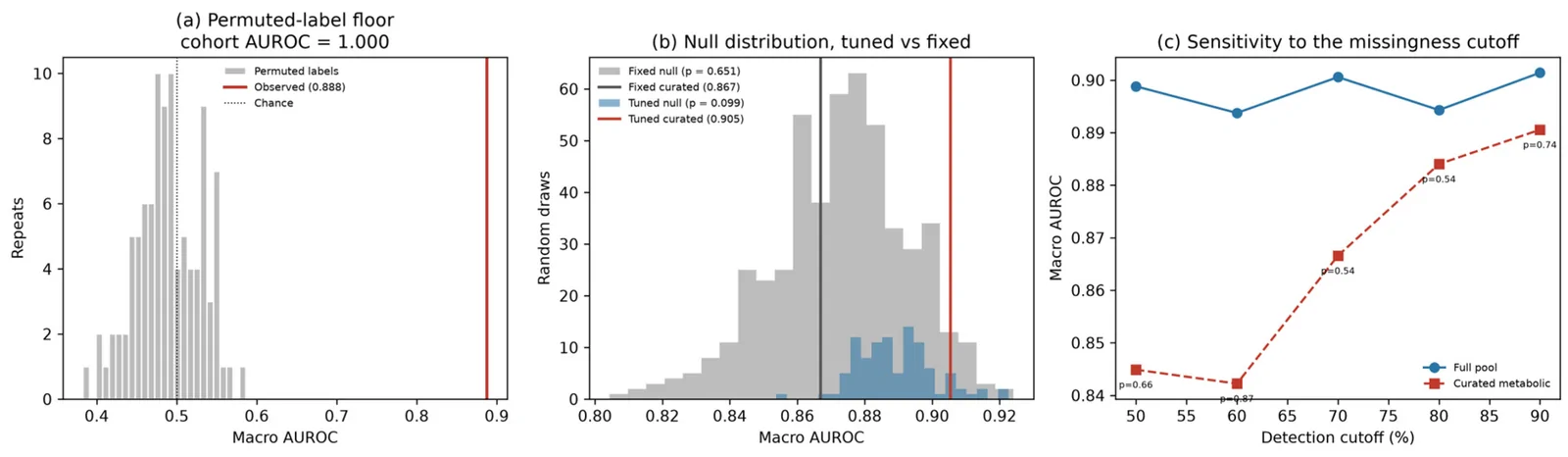
Sensitivity analyses for the classification and null comparisons. (**a**) Macro AUROC under 100 permutations of the subtype label, against the observed value; the dotted line marks chance (0.5). (**b**) Null distributions for the curated metabolic set under fixed and fully tuned hyperparameters. (**c**) Macro AUROC for the full pool and the curated set across protein detection cutoffs, with the empirical *p* value at each cutoff.

Magnitude of batch structure. Cohort membership was itself almost perfectly predictable from the proteome (AUROC 1.000), so the technical difference between the initial and expansion sets is larger than the principal-component summary in Section 3.1 conveys. Two further analyses bound its influence. Classifying a randomly permuted subtype label with the same pipeline gave a mean macro AUROC of 0.490 with a maximum of 0.587 over 100 permutations, against an observed 0.888, so the procedure does not manufacture performance (empirical *p* = 0.010; Figure 9a). Fitting and evaluating entirely within each cohort, where no between-cohort difference can contribute, gave 0.889 in the initial set and 0.867 in the expansion set, both close to the pooled 0.901. Subtype signal and batch structure coexist, and the classification result does not depend on the latter.

Tuned versus fixed hyperparameters in the null. The null distribution used fixed hyperparameters for tractability across repeated refits. Repeating it with full inner tuning in each of 100 draws gave a curated-set value of 0.9055 against a random mean of 0.8894 (empirical *p* = 0.099, z = +1.39), compared with 0.8670 against 0.8731 under fixed hyperparameters (*p* = 0.651, z = −0.30), against *p* = 0.649 and z = −0.33 in the primary run. Both procedures place the curated set inside the null distribution, and neither supports a privileged role for metabolic enzymes (Figure 9b).

Detection cutoff. The 70 percent detection requirement removes proteins that are quantified only in tumor tissue. Repeating classification and the metabolic comparison at cutoffs of 50, 60, 70, 80 and 90 percent gave macro AUROC values between 0.894 and 0.901 for the full pool and empirical *p* values between 0.54 and 0.87 for the curated set, with no cutoff bringing it outside the null (Table 6, Figure 9c). Adding the 2408 informative proteins excluded at the 70 percent cutoff back as binary detection indicators gave 0.887, slightly below the 0.901 obtained without them, so the discarded presence-absence information does not materially change classification.

## 4. Discussion

This study produced four main results. Proteomic classification of the CPTAC subtypes was accurate and held up when the model was locked before testing, and comparable accuracy was obtained for a label defined without any proteomic input, which limits how much of the result can be attributed to circularity. The subtypes did not materially improve prognostic models containing established clinicopathological variables, and confidence-stratified analyses provided no evidence that low-confidence subtype assignment was the primary explanation. Finally, a curated metabolic feature set classified no better than random sets of equal size, which constrains what the classification result can be taken to mean.

### 4.1. Subtype Information Is Redundant Rather than Localized

The macro AUROC values of 0.901 under cross-validation, 0.862 in the locked hold-out and 0.905 for the independently defined label indicate that the classifier assigns subtypes reproducibly. Calibration was acceptable for NMF3 and moderate for the other two classes.

These values do not, however, identify which proteins carry the subtype information. That inference requires a comparison against chance, and the metabolic set fell in the middle of the null distribution. The feature-count curve indicates why: with 211 samples, three classes and proteins that are extensively co-regulated, performance saturates at a few hundred randomly chosen features, above which feature identity no longer affects accuracy.

The housekeeping comparison qualifies this conclusion. Ribosomal, spliceosomal and proteasomal proteins scored significantly below a size-matched random draw, so the proteome is not uniform with respect to subtype information. Rather, the resolution of this design was sufficient to distinguish the machinery of protein synthesis from the bulk of the proteome but not to distinguish metabolic enzymes from it. The pattern is better described as redundant encoding across many correlated proteins than as concentration in a particular functional class.

This bears on a common analytical practice. Selecting a gene set on biological grounds, showing that it classifies or predicts well, and concluding that the selected genes are important does not hold up unless the set is also compared against random sets of the same size. The ccRCC signature studies cited here [19,20,21,22] follow this pattern, and we are not aware of published null-distribution comparisons for those panels. This does not establish that the signatures are meaningless; it indicates that the evidence usually offered in their support cannot discriminate a biologically privileged set from an arbitrary one of equal dimension.

### 4.2. Circularity, and What the Methylation Analysis Settles

Because the NMF labels were built by integrating several molecular layers, one of which was the proteome, a proteomic classifier is in part recovering its own input. The concern applies to the CPTAC subtype literature generally.

The methylation subtypes provide a direct check. They were defined from methylation data alone, so the proteome made no contribution to their construction, and predicting them from proteins involves no circularity. The macro AUROC was 0.905, matching the NMF result. Accuracy of this order is attainable without circular label construction in this cohort.

This does not establish that the NMF estimate is uninflated, since a shared underlying biology could drive both labels and both classifiers. It does, however, argue against the more specific concern that the model is recovering its own construction noise, which would not be expected to yield a result of equal magnitude on an independently constructed label.

### 4.3. Why NMF3 Separates and NMF1 Does Not

Classification performance differed markedly between classes, and the same pattern was seen in cross-validation and in both hold-out directions. NMF3 reached AUROC values between 0.959 and 0.988 with Brier scores of 0.039 to 0.065, whereas NMF1 was recognized poorly, with a sensitivity of 0.42 in one hold-out direction and 0.56 in the other, and was confused almost exclusively with NMF2.

Only 119 of the 212 cases annotated by Li et al. [16] were assigned to a core cluster, so nearly half lie near a cluster boundary. That denominator is the one used in the source publication and differs by one from our analysis cohort of 211, which excludes a single tumor without a usable proteome. Two explanations are consistent with this pattern: one class may be genuinely distinct while the other two are adjacent, or the labeling procedure may have produced one stable cluster and two unstable ones. Our data do not distinguish between them, although the core-membership annotation in the CPTAC table is relevant. Where one group behaves as a discrete category and two behave as ends of a gradient, describing the scheme in those terms may be more accurate than treating all three as equivalent. This bears directly on the downstream analyses. An unstable boundary between NMF1 and NMF2 limits what any prognostic comparison involving those classes can resolve, which is one reason the three-level contrast is reported alongside, rather than in place of, the NMF3 contrast. A scheme in which only one class is reliably recoverable is a weaker basis for clinical stratification than three well-separated classes would be, and the negative prognostic result should be read with that in mind.

### 4.4. Replication of Structure Without Incremental Prognostic Value

Subtype proportions in TCGA-KIRC came within 2.5 percentage points of CPTAC across a platform change, a cohort five times larger and a different patient population. This is transfer of structure, not validation of accuracy, since TCGA carries no ground-truth CPTAC labels. Within that limit it indicates that the subtype structure is a property of the disease and not an artifact of the training cohort.

The transferred subtypes showed no measurable incremental prognostic value over the clinical models evaluated here, which we assessed by nested model comparison rather than from a single hazard ratio. Entering subtype as a three-level categorical variable alongside age and stage produced a likelihood-ratio *p* of 0.314 on two degrees of freedom and a change in C-index of +0.0033, with neither class contrast approaching significance. Repeating this with Fuhrman grade in the model gave *p* = 0.703, and stratifying the baseline hazard by stage gave *p* = 0.705.

The comparison involving grade is the most informative of these. Grade is an established prognostic variable in ccRCC and behaved as expected here: fitted on the same 518 patients, adding grade to age and stage gave a likelihood-ratio *p* of 0.001 and a C-index gain of 0.0103, whereas adding subtype on top of grade gave *p* = 0.703 and a gain of 0.0019. An analysis that detected no prognostic signal at all might simply lack power, but one that detects grade and not subtype in identical models is making a comparative statement. If predicted rather than observed labels were attenuating a real association, the hazard ratio would be expected to move away from unity as assignment confidence increased; instead it moved toward unity, from 1.18 to 0.98. This argues against attenuation as the explanation, although confidence-based subsetting is itself a form of selection and cannot exclude it entirely.

The exploratory window analyses identified an apparent effect beyond four years (*p* = 0.003), which we report alongside the null result rather than in place of it. Those windows were not adjusted for stage, and the proportional hazards violation detected for stage itself makes differing stage composition across time windows the more parsimonious explanation. Reporting the late-window result in isolation would have supported a claim of a late-separating prognostic subtype that the full analysis does not sustain.

Our finding contrasts with a report from an independent Chinese proteogenomic cohort of 232 ccRCC cases, in which the most aggressive proteomic subtype remained an independent prognostic factor after adjustment for TNM stage and ISUP grade (HR 3.15, 1.59 to 6.21) [35]. Several differences between the two studies could account for this. Those subtypes were derived by consensus clustering of the proteome itself, and the aggressive group was defined largely by immune infiltration, which is not the construct measured by the CPTAC NMF scheme. Their labels were also assigned directly instead of being predicted, so the attenuation question does not arise, and the two populations differ in genomic background and clinical composition. Taken with our own results, this suggests that the prognostic content of a proteomic subtype depends on how the subtype was constructed rather than on its being proteomic, which supports evaluating clinical value separately from molecular coherence.

These results do not diminish the value of the CPTAC subtypes for their intended purpose. They were defined to describe molecular heterogeneity, which they do reproducibly, and prognosis was not among their stated aims. Stage and grade set a demanding benchmark in ccRCC, and validated tools such as the SSIGN score already combine them effectively [23]. Earlier evaluations of molecular subtypes against clinical variables have given mixed results. The ccA/ccB scheme separated survival when assigned from primary tumors but showed no significant association with outcome when assigned from metastatic tumors [13]. In a different setting, subtype assignment was reported to add to the International Metastatic RCC Database Consortium model [14].

### 4.5. Enzyme Abundance Is Not Metabolite Abundance

Our metabolic feature set was built from enzyme protein levels rather than measured metabolites, and enzyme abundance constrains flux without determining it. Transcriptomic and metabolomic views of the same ccRCC pathways are discordant [36], protein levels are shaped substantially by post-transcriptional regulation [37], and proteome-metabolome decoupling appears to follow from network constraint structure rather than measurement error [38].

Targeted metabolomics was available for 50 of the 211 tumors in the analysis cohort [16], which at 80 percent power detects correlations of roughly 0.39 and no smaller, so we do not report those analyses; matched measurement in 150 or more tumors would be needed to address the question. The metabolic phenotype of the cohort itself is described in Note S1 and Figure S4.

### 4.6. Dependency Data Cannot Support Functional Validation Here

We attempted to test whether subtype-discriminating proteins are functionally required, using CRISPR dependency data, but the available resource does not support the question in this disease. As a positive control, EPAS1, which encodes HIF-2 alpha and is the target of belzutifan [7,8], was only marginally more essential in kidney lines than elsewhere and passed the essentiality threshold in 17 percent of them. DepMap contains 42 screened kidney lines, of which 16 are annotated as clear cell, so this estimate rests on a small and poorly representative panel.

A panel that only weakly recovers the target of an approved drug for this disease cannot be used to exclude other targets. Most available kidney lines are VHL-competent and do not reproduce the constitutive HIF activation that defines the tumor. Tumor-anchored dependency maps now project essentiality onto patient tumors including clear cell renal cell carcinoma, although they are trained on the same cell line panels and inherit their coverage limits, and next-generation three-dimensional panels do not yet include renal lineages [39,40]. We report no target prioritization; the full analysis is given in Note S2.

### 4.7. Limitations

The initial and expansion sets separate along the third principal component, and all 11 differentially scored pathways shift in the same direction, which points to a technical origin. Cohort membership was not supplied to the classifier as a feature, so the hold-out results in Section 3.3 include whatever batch structure exists between the two sets; this is one of two explanations for the drop relative to pooled cross-validation and we cannot separate it from the smaller development sample.

Three further limitations follow from the analyses in Section 3.8. The two CPTAC cohorts are perfectly separable from the proteome, so technical structure in this dataset is substantial; within-cohort classification shows that the subtype result does not depend on it, but the pooled estimate cannot be regarded as free of it. The confidence interval for the subtype increment in discrimination extends to +0.0116, which does not exclude a gain of the size contributed by grade, so the negative prognostic finding is an upper bound on a small effect rather than a demonstration of none. And with 171 deaths the study could detect a hazard ratio of about 1.64 at conventional power, so a modest independent association remains compatible with these data and would require a substantially larger cohort to exclude.

Missingness in DIA proteomics is not random, and imputation strategy affects downstream inference [28,29,30]. Requiring quantification in 70 percent of samples removed 2591 proteins, among them tumor-restricted proteins such as NDUFA4L2, CA9 and CD70 that were absent from normal tissue entirely. Their exclusion biases the feature space toward abundant proteins.

The null-distribution comparison used fixed hyperparameters across the repeated refits, which lowered absolute performance by roughly 0.02 AUROC units relative to the tuned estimates. The comparison between feature sets is internally consistent, since all were evaluated identically, but the absolute values in Section 3.5 should not be compared directly against Table 2. For the 139-protein and 92-protein sets the fixed setting selected all available features, since the selection cap of 150 exceeded the set size; for the 3000-protein pool it selected 150.

The grade field in the GDC-harmonized TCGA-KIRC clinical table was empty for all patients, a known consequence of harmonization for this cohort, so Fuhrman grade was taken from the legacy TCGA release. This introduces a source inconsistency between grade and the other clinical variables, and grade was available for 518 of the 529 patients with survival data. Proportional hazards were violated for stage and for the binary subtype contrast; we addressed this with stage-stratified models, non-overlapping time windows and a time-varying coefficient model (Section 3.8). Follow-up is short relative to the natural history of ccRCC, and the 173 deaths recorded in the 529-patient survival cohort limit what any survival model can resolve. TCGA subtypes were assigned by a classifier and not observed directly, a limitation we examined through the confidence-stratified analysis but cannot remove. Finally, the analysis is retrospective and uses public data throughout. The classifier has not been locked to a fixed protein list with defined cutoffs, which would be a prerequisite for clinical evaluation.

## 5. Conclusions

Proteomic data classify the CPTAC multi-omic subtypes of clear cell renal cell carcinoma accurately, and this holds in a locked hold-out design and for a methylation-derived label defined without proteomic input. A transcriptome version of the classifier reproduced subtype proportions in 533 independent TCGA-KIRC tumors to within 2.5 percentage points. Adding the predicted subtypes as a three-level variable did not improve a survival model containing age and stage, nor one that also contained Fuhrman grade, while grade itself remained prognostic in the same model; confidence-stratified analyses gave no evidence that this null result was driven by low-confidence subtype assignment. A curated set of 139 metabolic enzymes classified subtypes no better than random sets of the same size, or than random sets matched on abundance, missingness, variance and co-regulation, whereas a size-matched housekeeping set performed significantly worse. Subtype information is therefore spread broadly and redundantly across the proteome; it is not concentrated in a functional class. For clinical purposes, a molecular subtype scheme in ccRCC should be evaluated against stage and grade, not against an unadjusted model, and studies that select a gene set on biological grounds should report a comparison against random sets of equal size.

## Figures and Tables

**Table 1:** Analysis cohort characteristics and data availability by molecular layer.

Characteristic	*n* (%)
Total tumors analyzed	211
CPTAC initial set	103 (48.8)
CPTAC expansion set	108 (51.2)
NMF consensus subtype	
NMF1	58 (27.5)
NMF2	103 (48.8)
NMF3	50 (23.7)
Histologic subtype	
Conventional low grade (CL)	119 (56.4)
Conventional high grade (CH)	75 (35.5)
CH with sarcomatoid features	14 (6.6)
CH with rhabdoid features	3 (1.4)
Tumor grade	
G1	17 (8.1)
G2	102 (48.3)
G3	63 (29.9)
G4	29 (13.7)
Tumor stage	
I	96 (45.5)
II	21 (10.0)
III	56 (26.5)
IV	21 (10.0)
Not reported	17 (8.1)
Molecular layers available	
Proteome (5771 proteins)	211 (100)
Matched adjacent normal tissue	166 (78.7)
Transcriptome	103 (48.8)
Targeted metabolome (183 metabolites)	50 (23.7)
Outcome data	
Recurrence recorded	211 (100), 36 events
Survival recorded	194 (91.9), 30 deaths

Percentages are of the 211 tumors in the analysis cohort, and every category sums to 211. CL, conventional low grade; CH, conventional high grade; CH-S, CH with sarcomatoid features; CH-R, CH with rhabdoid features. Overall survival was not recorded for 17 tumors; recurrence status was available for all 211.

**Table 2:** Classification performance under nested cross-validation.

Feature Set	Model	Macro AUROC (95% CI)	Balanced Accuracy	AUROC NMF1	AUROC NMF2	AUROC NMF3
Full proteome (3000)	Logistic regression (L2)	0.901 (0.871–0.933)	0.754	0.837	0.889	0.977
Full proteome (3000)	SVM (RBF)	0.894 (0.860–0.927)	0.767	0.827	0.887	0.967
Full proteome (3000)	Extra trees	0.889 (0.857–0.923)	0.728	0.826	0.877	0.964
Full proteome (3000)	Random forest	0.887 (0.849–0.920)	0.751	0.825	0.873	0.962
Full proteome (3000)	Linear discriminant analysis	0.871 (0.835–0.913)	0.739	0.810	0.849	0.954
Metabolic subset (139)	Logistic regression (L2)	0.905 (0.878–0.934)	0.730	0.877	0.893	0.946

All values are computed from outer-fold predictions. CI, percentile bootstrap confidence interval over 300 resamples.

**Table 3:** Locked hold-out validation. Development includes all tuning; the locked model was applied once to the test cohort.

Development	Test	Macro AUROC (95% CI)	Balanced Accuracy	AUROC NMF1	AUROC NMF2	AUROC NMF3
Initial (*n* = 103)	Expansion (*n* = 108)	0.862 (0.802–0.914)	0.700	0.788	0.839	0.959
Expansion (*n* = 108)	Initial (*n* = 103)	0.894 (0.848–0.937)	0.769	0.826	0.869	0.988

**Table 4:** Nested Cox proportional hazards models for overall survival in TCGA-KIRC, all fitted on the same 518 patients with 171 deaths for whom age, stage and Fuhrman grade were available. Subtype enters as a three-level categorical variable with NMF1 as reference.

Model	Covariate	Hazard Ratio	95% CI	*p*	C-Index
A. Age + stage	Age (per year)	1.03	1.01–1.04	0.0001	0.7314
	Stage III–IV	3.51	2.52–4.89	<0.0001	
B. A + grade	Age (per year)	1.03	1.01–1.04	0.0001	0.7418
	Stage III–IV	3.14	2.24–4.39	<0.0001	
	Grade G3–G4	1.75	1.22–2.52	0.0026	
C. B + subtype	Age (per year)	1.03	1.01–1.04	0.0001	0.7437
	Stage III–IV	3.12	2.23–4.37	<0.0001	
	Grade G3–G4	1.74	1.21–2.51	0.0029	
	NMF2 (vs. NMF1)	0.88	0.61–1.27	0.50	
	NMF3 (vs. NMF1)	1.01	0.68–1.49	0.96	

Likelihood-ratio tests: grade over A, chi-square 10.766, df 1, *p* = 0.001, change in C-index +0.0103; subtype over B, chi-square 0.705, df 2, *p* = 0.703, change in C-index +0.0019. Changes in C-index were computed on unrounded values and may differ in the last digit from the difference of the rounded values shown in the table.

**Table 5:** Sensitivity of the incremental prognostic analysis to modeling choices. All models were fitted on the same 518 patients with 171 deaths. Subtype enters as a three-level categorical variable with NMF1 as reference except where stated.

Model Specification	Clinical C-Index	Plus Subtype	LR chi-Square (df 2)	*p*	Change in C-Index
Stage and grade dichotomized (primary)	0.7418	0.7437	0.705	0.703	+0.0019
Stage and grade as ordinal scores	0.7638	0.7643	0.422	0.810	+0.0004
Stage and grade as four-level factors	0.7686	0.7694	0.640	0.726	+0.0008
Stage-and-grade composite score	0.7638	0.7647	0.668	0.716	+0.0009
Continuous class probabilities	0.7418	0.7433	0.516	0.773	+0.0015
Stage-stratified baseline hazard	—	—	0.700	0.705	—
Time-varying subtype coefficient	—	—	1.878	0.391	—

**Table 6:** Sensitivity of classification and of the metabolic-versus-random comparison to the protein detection cutoff. The analysis pool was capped at the 3000 most variable proteins at every cutoff.

Detection Cutoff	Proteins Retained	Full Pool Macro AUROC	Curated Metabolic	Empirical *p*
50%	6495	0.8988	0.8449	0.656
60%	6142	0.8938	0.8422	0.869
70% (primary)	5755	0.9006	0.8666	0.541
80%	5294	0.8943	0.8840	0.541
90%	4662	0.9014	0.8906	0.738

## Data Availability

All data analyzed in this study are publicly available. CPTAC ccRCC proteomic data were obtained from the Proteomic Data Commons (accession PDC000411); consensus subtype labels, clinical annotation and targeted metabolomics were taken from Supplementary Tables S1 and S7 of the source publication [16]; CPTAC transcriptomic data were obtained through the cptac Python package. TCGA-KIRC STAR-TPM expression, survival, GDC-harmonized clinical and legacy clinical matrices, together with the gencode v36 probemap, were downloaded from the UCSC Xena platform. CRISPR gene effect scores and cell line annotation were downloaded from the DepMap portal. A source data inventory listing every file, its release and its SHA-256 checksum is provided as Table S1. All analysis code, including the scripts that generated every figure and table, is archived on Zenodo under DOI 10.5281/zenodo.22660275 (https://doi.org/10.5281/zenodo.22660275). The deposit contains the full analysis pipeline and the random seeds needed to reproduce the reported values from the public source data.

## Supplementary Materials

The following supporting information can be downloaded at: https://www.mdpi.com/article/10.3390/life16101752/s1, Figure S1: SHAP importance for the top 25 proteins in the full-proteome model; Figure S2: Quality control, comprising the missing value profile, cohort structure in principal component space, the tumor against matched normal volcano plot and the VHL-HIF positive control; Figure S3: Secondary analyses of the binary NMF3-against-remainder contrast, comprising confidence-stratified hazard ratios and log-rank *p* values within non-overlapping follow-up windows; Figure S4: Metabolic pathway reprogramming across 166 matched tumor and normal pairs; Note S1: Metabolic reprogramming in tumor against matched normal tissue; Note S2: CRISPR dependency screening; Table S1: Source data inventory listing every primary data file, its release and its SHA-256 checksum.
