# Supplementary material for: CPTAC Consensus Subtypes Add No Incremental Prognostic Value Beyond Stage and Grade in Clear Cell Renal Cell Carcinoma: A Proteomic Reanalysis
Source: Life (Basel). 2026 Oct 19;16(10):1752. doi: 10.3390/life16101752 (PMC13705993; doi:10.3390/life16101752)
Supplement: Supplementary file 1 [file life-16-01752-s001.zip › life-4586686-supplementary.pdf]

Supplementary Materials

CPTAC Consensus Subtypes Add No Incremental Prognostic Value Beyond Stage and Grade in Clear Cell Renal Cell Carcinoma: A Proteomic Reanalysis

Seyma Yasar, Fatma Hilal Yagin, Sarah A. Alzakari, Amal K. Alkhalifa, Fahaid Al-Hashem and Abedelmalek Kalefh Tabnjh

Life 2026

Contents

Figure S1. SHAP importance for the top 25 proteins in the full-proteome model

Figure S2. Quality control

Figure S3. Secondary analyses of the binary NMF3-against-remainder contrast

Note S1. Metabolic reprogramming in tumor against matched normal tissue (with Figure S4)

Figure S4. Metabolic pathway reprogramming across 166 matched tumor and normal pairs

Note S2. CRISPR dependency screening

Table S1. Source data inventory

Reference numbers in square brackets refer to the reference list of the main manuscript.

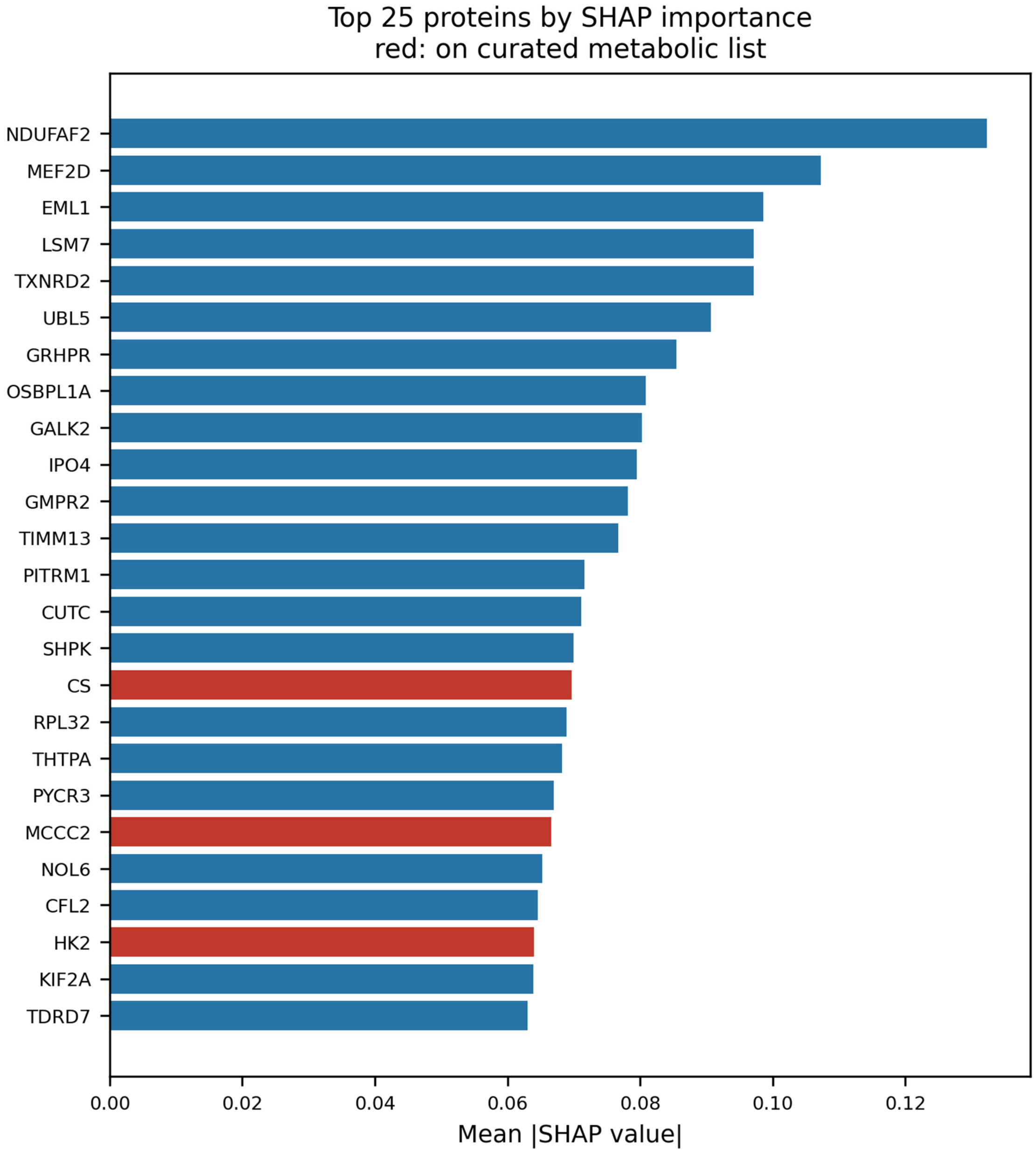

Figure S1. SHAP importance for the top 25 proteins in the full-proteome model. Bars show the mean absolute SHAP value from the L2-penalized logistic regression model refitted on the full analysis cohort; red bars mark proteins that belong to the curated metabolic set. SHAP values describe the fitted model and are not a performance estimate.

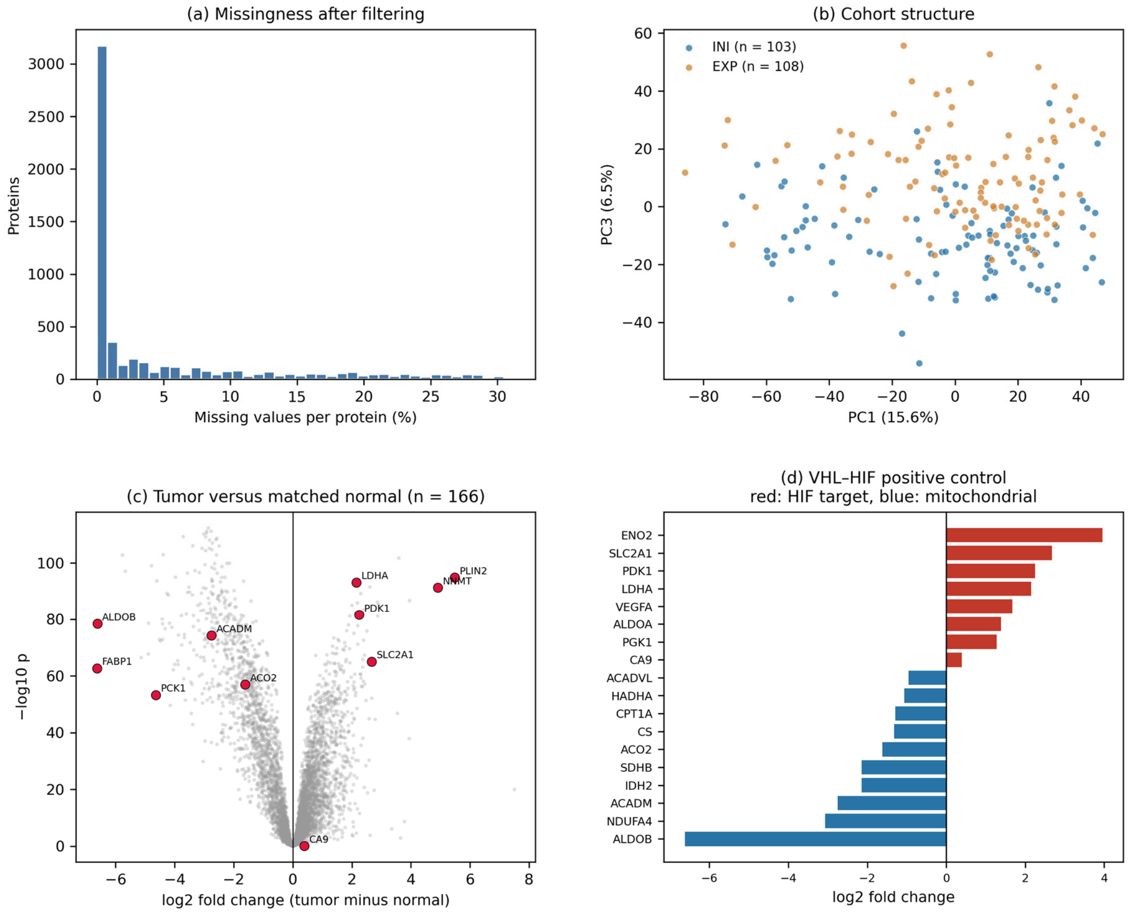

Figure S2. Quality control. (a) Missing value profile per protein after the 70 percent detection filter. (b) Cohort structure in principal component space (PC1 against PC3); the initial (INI) and expansion (EXP) sets separate along PC3. (c) Tumor against matched normal volcano plot from the paired t-test across 166 pairs, with selected proteins highlighted. (d) VHL–HIF positive control: log2 fold change of canonical HIF targets (red) and mitochondrial enzymes (blue).

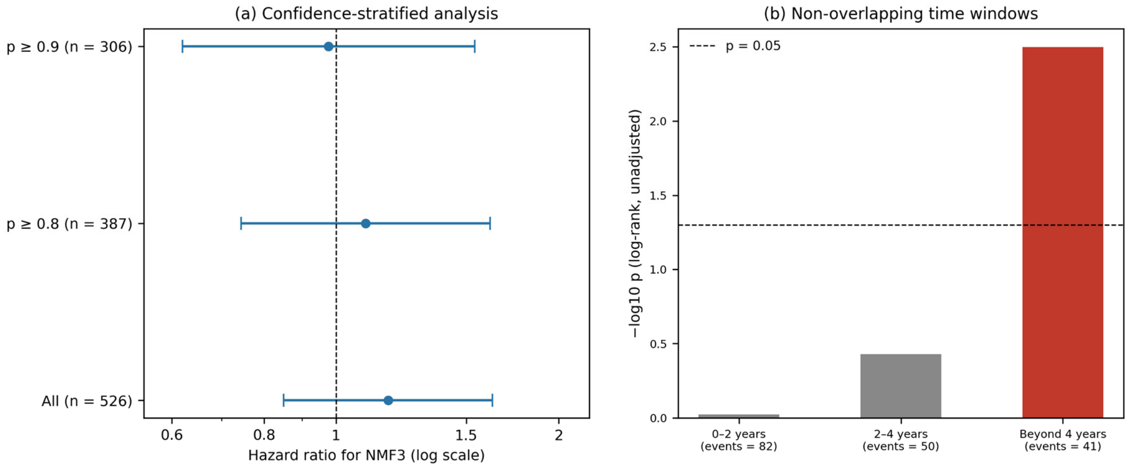

Figure S3. Secondary analyses of the binary NMF3-against-remainder contrast. (a) Hazard ratios for NMF3 from Cox models containing age and stage, in all patients and in subsets with assignment probability of at least 0.8 and 0.9; the dashed line marks a hazard ratio of 1. (b) Log-rank p values within non-overlapping follow-up windows, unadjusted for stage; the dashed line marks p = 0.05.

Note S1. Metabolic reprogramming in tumor against matched normal tissue

This analysis characterizes the metabolic phenotype of the cohort. It is established background for clear cell renal cell carcinoma rather than a finding of the present study, and it is not used to support any conclusion in the main text. The pathway scoring procedure is described in Section 2.3 of the main manuscript.

Comparing the 166 matched pairs, 4966 of 5771 proteins differed at FDR below 0.05, and 1397 of those changed by more than twofold. The proteins lost in tumor were dominated by proximal tubule metabolism: FABP1 (log2FC −6.63), ALDOB (−6.62), ACSF2 (−5.76), ABAT (−5.67), ALDH6A1 (−4.78) and the gluconeogenic carboxykinases PCK1 (−4.64) and PCK2 (−4.61). Proteins gained included FABP7 (+7.51), the lipid droplet coat protein PLIN2 (+5.48), NNMT (+4.91), a metabolic enzyme proposed as a drug target in ccRCC [6], ENO2 (+3.95) and the lactate exporter SLC16A3 (+3.94).

Aggregating to 22 curated metabolic pathways reproduced the expected direction of the VHL–HIF axis without supervision (Figure S4), matching the pattern described in transcriptomic and metabolomic surveys of the same disease [3,24,36]. Complex I of oxidative phosphorylation fell furthest (d = −6.15), followed by complex III/V (−4.21), branched-chain amino acid catabolism (−4.13), the TCA cycle (−3.76) and fatty acid oxidation (−3.01). HIF target proteins rose most (+4.07), then glycolysis (+3.65) and lipid droplet proteins (+3.25).

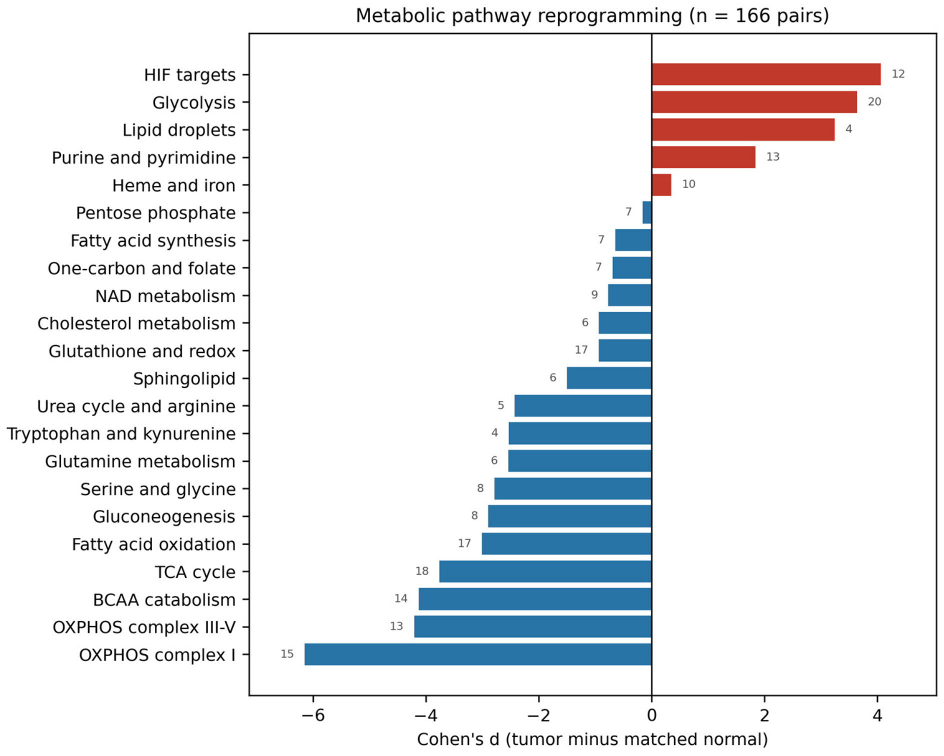

Figure S4. Metabolic pathway reprogramming across 166 matched tumor and normal pairs. Bars show Cohen’s d for each of 22 curated pathway scores (tumor minus matched normal); red indicates higher in tumor. Numbers next to the bars give the quantified proteins per pathway.

A group of proteins escaped the paired comparison because they were absent from normal tissue rather than merely reduced. NDUFA4L2 was quantified in 94.1 percent of tumors and 0 percent of normals, CA9 in 95.0

against 1.2 percent, and CD70 in 75.1 against 0 percent. The reverse pattern identified markers of nephron segments the tumor does not arise from: CASR (0.9 against 98.8 percent), ATP6V1G3 (2.3 against 99.4), CLDN10 (4.1 against 100) and BSND (1.4 against 96.5). These are distal tubule and collecting duct proteins, consistent with the proximal tubule origin of ccRCC.

Note S2. CRISPR dependency screening

Full account of the analysis summarized in Section 4.6 of the main manuscript. No target prioritization is derived from it.

Cell line selection. The DepMap model annotation lists 87 lines assigned to a kidney or renal lineage, of which 42 carry genome-scale CRISPR screens. This lineage definition is broader than renal carcinoma alone: it includes Wilms tumor, extrarenal rhabdoid tumor, immortalized embryonic kidney cells and a small number of lines assigned to other primary diseases. Sixteen of the 42 screened lines are annotated as renal clear cell carcinoma, and this subset is stable across alternative lineage definitions. Gene effect scores were taken from the Chronos model [26].

Positive controls. Six genes with established relevance to clear cell renal cell carcinoma were tested for kidney-selective essentiality: EPAS1, ARNT, VHL, HIF1A, VEGFA and CCND1. Only EPAS1 crossed the significance threshold, with a difference of 0.14 in mean Chronos score ($p = 0.025$), and it passed the conventional essentiality threshold in 17 percent of kidney lines. ARNT, the obligate partner of HIF-2 alpha, showed no signal ($p = 0.70$). VHL ran in the opposite direction, which is expected for a loss-of-function tumor suppressor and means it is not a valid positive control in this setting.

Interpretation. A resource that recovers the target of an approved drug for this disease only weakly and partially, and that gives no signal for the obligate partner of that target, does not have the resolution to support or exclude selectivity claims. We therefore treat the absence of kidney-selective dependencies among subtype-discriminating proteins as a limitation of the resource rather than as a finding. Most available kidney lines are VHL-competent and do not reproduce the constitutive HIF activation that defines the tumor. No target prioritization is reported.

A note on an unreported comparison. Subtype-discriminating proteins had a lower mean Chronos score than genes overall (−0.334 against −0.142, $p = 0.0008$), which could be read as evidence of functional relevance. We do not report this in the main text because it is attributable to composition rather than biology. Ribosomal, spliceosomal and proteasomal proteins make up 17 of the 141 proteins tested (12 percent), yet they account for 15 of the 24 genes (62 percent) that reach the essentiality threshold. Housekeeping genes are essential in effectively every cell line, so a panel containing even a modest fraction of them will appear more essential than genes overall regardless of any subtype-specific dependency.

Abundance bias check. Feature importance in the classifier was not associated with protein abundance (Spearman $\rho = -0.042$, $p = 0.61$), indicating that the ranking is not driven by how well proteins are measured (Figure S1).

Table S1. Source data inventory

Every primary data file used in the analysis, its source and its SHA-256 checksum, followed by a reconciliation of the sample counts reported in the manuscript. Checksums were computed on the files as downloaded; the analysis code that consumes them is archived on Zenodo (https://doi.org/10.5281/zenodo.22660275).

File	Source	Used for	SHA-256
CPTAC_ccRCC_global_proteome_DIA.tsv	Proteomic Data Commons, study PDC000411 (publication supplementary archive, PMID 36563681)	DIA global proteome, tumor and adjacent normal specimens	35b6af2ebdc6d71f3108496c1997e4773b61aa24c0de8a81122a151417fe4782
ccrcc_clinical_TableS1.xlsx	Li et al. 2023 [16], Supplementary Table S1 (Meta_table)	Clinical annotation, NMF consensus / methylation / immune subtypes, outcome data	3407c020b559dda3c4006c39cbb61188062244c65c6b6c8f3cf94fcfbe9bee39
cptac_transcriptomics.parquet	cptac Python package (CPTAC ccRCC RNA-seq)	Transcriptome for the cross-platform classifier	14a1323e8a47165d00ac9e1c8aa2765be6a9f3d2d8295155a1aaf67017fa224e
ccrcc_metabolomics_supp.xlsx	Li et al. 2023 [16], Supplementary Table S7	Targeted metabolomics (183 metabolites; described only)	191206c7c41819f877dd1f06a3ac5874e7a1fd5182dfcb2a8c8fd0fc2d053b8c
TCGA-KIRC.star_tpm.tsv.gz	UCSC Xena [25], GDC TCGA-KIRC hub	STAR-TPM expression, 533 primary tumors	70ab9bb0faa27cc76003626546c2f87658606a240781f591552d88176de3c3b9
TCGA-KIRC.survival.tsv.gz	UCSC Xena [25], GDC TCGA-KIRC hub	Overall survival	504fc3ff66b46e01423dafef423e1e5db87a2e9f052035b13907f997cf8e491c
TCGA-KIRC.clinical.tsv.gz	UCSC Xena [25], GDC-harmonized clinical table	Age, stage	0ad39ca22e50264cdcd8f2b6fc386f44eefd0c3e4794fea5799587e96689271b
TCGA-KIRC.legacy_clinical.tsv (legacy clinical matrix)	UCSC Xena [25], TCGA hub (legacy)	Fuhrman grade (empty in the GDC-harmonized release)	2684f28e57ace0afc4b2d405be532d57337f004e0683df07f651bc19cb59d294
gencode.v36 probemap	UCSC Xena [25], ID/Gene mapping	Ensembl identifier to gene symbol	f027752d663b1da74f4113998bf008e6ebecfa3732607513e027bb6eb1f635b5
CRISPRGeneEffect.csv	DepMap portal	Chronos gene effect scores	e610a4cefb13a82b5b256b47eb08b63ff14843f8dbd0fb164bc0a32688e5b89e
Model.csv	DepMap portal	Cell line annotation (lineage, OncoTree subtype)	ea4e0b2a3bc806f81df62689a5ae75f1a100135727a3d7b8a4c7ccc8815183f8

Sample-count reconciliation

Quantity	Count
CPTAC confirmatory cohort, cases	213
DIA proteome, tumor / adjacent normal specimens	222 / 171
Analysis cohort (tumors with an NMF consensus label, usable proteome)	211
Matched tumor–normal pairs in the analysis cohort	166
Analysis-cohort tumors with transcriptome / targeted metabolome	103 / 50
Proteins retained at the 70 percent detection cutoff	5771
TCGA-KIRC primary tumors with expression	533
TCGA-KIRC with survival data (deaths)	529 (173)
TCGA-KIRC with age and stage (deaths)	526 (172)
TCGA-KIRC with age, stage and Fuhrman grade (deaths)	518 (171)
DepMap kidney-lineage lines with CRISPR screens / annotated clear cell	42 / 16
